# Gestational exposure to particulate matter from urban wildfires is associated with changes in circulating oxylipins but not flame retardants 7 to 13 months post-exposure

**DOI:** 10.1016/j.envint.2025.109468

**Published:** 2025-04-20

**Authors:** Eunyoung Park, Qing Shen, Zhichao Zhang, Claire E. O’Brien, Amanda J. Goodrich, Elizabeth E. Angel, Irva Hertz-Picciotto, Daniel J. Tancredi, Sean Raffuse, Deborah H. Bennett, Rebecca J. Schmidt, Ameer Y. Taha

**Affiliations:** a Department of Food Science and Technology, University of California Davis, Davis, CA, USA; b Department of Public Health Sciences, School of Medicine, University of California Davis, Sacramento, CA, USA; c Department of Pediatrics, University of California Davis, Sacramento, CA, USA; d MIND Institute, University of California Davis, Sacramento 95817 CA, USA; e Air Quality Research Center, University of California Davis, Davis, CA, USA; f West Coast Metabolomics Center, Genome Center, University of California Davis, Davis, CA, USA; g Center for Neuroscience, University of California Davis, Davis, CA, USA

**Keywords:** Lipid mediators, Flame retardants, PAH, Wildfire, Particulate matter, Immune response

## Abstract

Exposure to fine particulate matter (PM_2.5_) from wildfire smoke has been linked to immune dysregulation underlying multiple health conditions, but data on the long-term effects of these exposures during gestation are lacking. Smoke PM_2.5_ from wildfires occurring in urban areas is of particular concern because it can carry persistent chemicals within household furniture or soil, as well as polyaromatic hydrocarbons (PAHs) from combusted materials. The present study investigated the long-term associations between wildfire PM_2.5_ and serum polybrominated diphenyl ethers (PBDEs), polychlorinated biphenyls (PCBs), PAHs and lipid mediators (i.e., oxylipins) involved in immune regulation in participants from the B-SAFE (Bio-Specimen Assessment of Fire Effects) study, which enrolled women pregnant during or shortly after the 2017 Tubbs Fire in California (n = 140). Serum samples were collected and assayed 7 to 13 months post-exposure, at which point 20 women were still pregnant and 120 women were postpartum. Adjusted linear regression models revealed a significant positive association between increasing PM_2.5_ (μg/m^3^) exposure and serum concentrations of benzo[k]fluoranthene, a PAH (β = 0.866, *P* = 0.0403, [95 %CI: 0.0389, 1.69]). No significant associations were observed between PM_2.5_ exposure and serum PBDEs, PCBs or other PAHs. Increased exposure to PM_2.5_ was associated with lower serum concentrations of lipoxygenase (LOX)-derived free oxylipins and increased concentrations of LOX-derived oxylipins esterified to circulating lipids. These findings provide new evidence of long-term effects of gestational wildfire PM_2.5_ exposure on serum benzo[k]fluoranthene levels and the turnover of oxylipins involved in immunity via the LOX pathway. Additional studies are warranted to better understand the impact of these changes on maternal and child health.

## 1. Introduction

Climate change has been linked to increased frequency and intensity of wildfires in both forested and urbanized areas (Xu et al. 2020). Wildfires generate particulate matter (PM), carbon monoxide, nitrogen oxide, ozone, and volatile organic compounds (Gill et al. 2024; Tao et al. 2020) that can be carried in smoke plumes for hundreds of kilometers away from the location of the fire (Black et al. 2017b; Reid et al. 2016).

Urban wildfires may pose greater risks to human health than forest wildfires due to the volatilization of flame retardants such as polybrominated diphenyl ethers (PBDEs) from household furniture and electronics, and environmental contaminants such as polychlorinated biphenyls (PCBs), and the generation of polyaromatic hydrocarbons (PAHs) from combusted household and structural materials. PBDEs, PCBs and PAHs can adsorb to PM (e.g. PM_2.5_), which can be carried by air currents to populated areas several kilometers from the fire center (Niemi et al. 2005; Wegesser Teresa et al. 2009). This is evidenced by studies showing the presence of PBDEs, PCBs and PAHs in air and soil collected several km from burn pit sites of combusted household furniture and electronics (Boström et al. 2002; Costa and Giordano 2014; Farrar et al. 2004; Meharg et al. 1998; Solorzano-Ochoa et al. 2012).

PM_2.5_ from wildfires has been associated with more adverse health outcomes compared to other types of PM_2.5_ exposure (Reid Colleen et al. 2016; Wegesser Teresa et al. 2009; Zhang et al. 2023). For instance, one study showed that PM_2.5_ derived from wildfires was associated with more hospitalizations compared to PM_2.5_ derived from non-wildfire sources (Aguilera et al. 2021). PM_2.5_ derived from wildfires was also shown to be associated with increased risk of respiratory tract infections in humans, and to induce pulmonary macrophage activation in mice, compared to non-wildfire PM_2.5_ (Aguilera et al. 2021; Migliaccio et al. 2013). Overall, these studies suggest that wildfire PM_2.5_ alters immune responses. While the majority of studies have looked at wildfire PM effects on the risk of cancer, respiratory disorders, dementia and overall mortality (Reid Colleen et al. 2016; Wegesser Teresa et al. 2009; Zhang et al. 2023), data on the long-term effects of wildfire PM exposures during gestation and related immune mechanisms are lacking.

PM_2.5_ components such as PBDEs, PCBs and PAHs are thought to promote immune dysregulation underlying multiple health conditions by altering the turnover of bioactive lipid mediators (i.e. oxylipins) derived from polyunsaturated fatty acid (PUFA) enzymatic oxidation via cyclooxygenase (COX), lipoxygenase (LOX), cytochrome P450 (CYP), and soluble epoxide hydrolase (sEH). These enzymes are directly coupled to cytokines and chemokines involved in inflammation and the resolution of inflammation (Dyall et al. 2022; Gabbs et al. 2015). Exposure to PM_2.5_ components such as PAHs have been shown to increase the production of arachidonic acid (AA)-derived lipid mediators involved in promoting inflammation (Lin et al. 2022). Additionally, exposure to non-wildfire PM_2.5_ during the second trimester of pregnancy was associated with increased cord blood concentrations of pro-inflammatory lipid mediators produced by the LOX pathway (Martens Dries et al. 2017). Although this study reflects specific effects of intrauterine non-wildfire PM_2.5_ exposure on postpartum inflammation at birth, the effects of PM_2.5_ originating from wildfires are not known.

To our knowledge, there are no human studies on the long-term effects of prenatal wildfire smoke exposure, particularly from urban fires where PBDEs, PCBs and PAHs may concentrate in smoke PM, on lipid-mediated immunity. Thus, the present study investigated the long-term associations between gestational wildfire PM exposure and serum PBDEs, PCBs, PAHs, oxylipins involved in immune regulation and PUFA precursors to oxylipins, in a subset of participants from the B-SAFE (Bio-Specimen Assessment of Fire Effects) study who were pregnant during or shortly after the 2017 Tubbs Fire in California. B-SAFE collected biospecimens and survey information to be used to understand biological responses and health impacts of gestational wildfire exposures. Samples were collected from mothers 7 to 13 months post-exposure, and assayed for chemicals (PBDEs, PCBs and PAHs), fatty acids, and oxylipins in both the free bioactive pool and the total lipid pool containing esterified oxylipins. The esterified pool constitutes the majority (~90 %) of circulating oxylipins in humans (Shearer and Newman 2008), and although it is less bioactive, it has recently been shown to serve as a major source and regulator of free bioactive oxylipin levels in plasma and tissues (Otoki et al. 2020; Taha et al. 2018). We hypothesized that gestational exposure to PM_2.5_ from fire plumes would be associated with long-lasting changes in oxylipins involved in inflammation-resolution by increasing blood levels of long-lived chemicals (e.g. PBDEs and PCBs (Idowu et al. 2023; Thuresson et al. 2006)).

## 2. Methods

### 2.1. Study cohort

The B-SAFE study enrolled women pregnant during or shortly after major wildfire events in California, and their children. A total of 172 mother–child pairs were enrolled in B-SAFE during the 2017 Napa/Sonoma (Tubbs) wildfires that occurred in urban areas within the Napa-Sanoma County regions. Women were eligible to participate if they were at least 18 years of age, resided in Northern California at the start of the wildfires, were able to speak, read, and understand English or Spanish. Participants were recruited primarily through local and social media campaigns and community outreach. Participants completed online or paper questionnaires collecting information on wildfire experiences, exposures, address history, pregnancy and health history, basic lifestyle and demographic factors, and pregnancy outcomes. The present analysis was performed in serum of 140 participants who provided blood 7 to 13 months after the Tubbs fire. Of the 140 subjects included in this study, 120 were postpartum and 20 were pregnant at the time of blood collection (Sup. Fig. 1). Serum samples were stored in plastic cryovials at −80 °C until the time of analysis in April of 2021. All samples (n = 140) were subjected to PBDE, PCB, PAH, lipid and oxylipin analysis.

### 2.2. PM_2.5_ measurement

Fine particulate matter (smaller than 2.5 μm in aerodynamic width, PM_2.5_) was estimated using models based on a smoke forecasting system that predicted near-surface PM_2.5_ concentrations (O’Neill et al. 2021; Raffuse et al. 2024). Customized smoke modeling products were constructed based on GOES-16 satellite fire detections. PM_2.5_ was monitored with Environmental Beta Attenuation Monitors (EBAM; Met One Instruments, Inc.) (Trent 2006). Estimated PM_2.5_ (μg/m^3^) by day was then linked to home and evacuation addresses reported by mothers during the wildfires. This was achieved by asking mothers for the addresses they evacuated to and the dates they were at that location, and assigning exposures based on geolocated address history. The spatial resolution of the modeled PM_2.5_ estimates was 4 km, which allowed for geographically specific exposure assignment based on each participant’s reported address. For each mother, average PM_2.5_ (μg/m^3^) was calculated during the peak exposure period, between October 9 and 10 of 2017, as this captured the highest recorded concentrations within the wildfire event. This method ensures a more accurate representation of exposure rather than relying on intermittent peaks. Notably, the wind direction was not steady throughout the exposure period, meaning all participants were likely directly exposed at some point. Given this variability, a strict classification of upstream versus downstream exposure was not feasible.

### 2.3. Extraction of PBDEs, PCBs and PAHs from Serum

The extraction of PBDEs, PCBs, and PAHs from serum was carried out using a validated method described by Lin et al. (Lin et al. 2013) as detailed in the Supplementary Methods. In brief, an aliquot of 0.5 mL serum was added into a disposable glass tube on ice, spiked with 10 μL of surrogate standard mix solution containing 500 ng/mL ^13^C_12_-PCB-11, ^13^C_12_-PCB-97, ^13^C_12_-BDE-28, ^13^C_12_-BDE-118, and D_12_-Chrysene in isooctane (AccuStandard, Inc., New Haven, CT, USA), and mixed with 0.5 mL of formic acid. The mixture was ultrasonicated for 10 min, and then applied to Oasis HLB solid phase extraction (SPE) columns. The extracts were dried under nitrogen, reconstituted in 100 μL of isooctane fortified with 25 ng/mL mirex, sonicated for 2 min, and transferred into GC/MS vials with inserts for gas chromatography-electron ionizationtandem mass spectrometry (GC-EI-MS/MS) analysis.

### 2.4. Extraction of total lipids (Fatty Acids and Cholesterol) and total oxylipins from serum

Total lipids were extracted from human serum samples using a modified version of the Folch method (Folch et al. 1957). Serum samples stored at −80 °C were thawed on ice for 1.5–2 h before extraction. An aliquot of 250 μL serum was added into an 8 mL glass tube containing 350 μL of chilled 1 mM 2Na-EDTA/0.9 % KCl and 2.4 mL of chloroform/methanol (2:1 v/v) with 0.002 % BHT. The bottom chloroform layer was extracted, and a second chloroform extraction was applied to retrieve the remaining lipids from the original glass tube. The chloroform layers were pooled, and reconstituted in 2 mL of chloroform/methanol (2:1 v/v), vortexed, transferred to 2 mL GC amber vials, and stored at −80 °C until further use.

A portion (1 mL) of the 2 mL Folch extracts was used for total fatty acid and cholesterol determination. Samples were spiked with 5-α cholestane and di17:0PC as internal standards, and transesterified with methanolic HCl as detailed in the Supplementary Methods. The transesterified fatty acid methyl esters and cholesterol were reconstituted in 100 μL of hexane stored in a −80 °C freezer until analysis.

Total (mainly esterified) oxylipin analysis was performed on the remaining 1 mL portion of the 2 mL Folch extract as detailed in Supplementary Methods. Samples were hydrolyzed in 0.4 M sodium hydroxide in methanol/water (1:1 v/v), and the hydrolyzed oxylipins were purified using 60 mg Oasis HLB 3 cc SPE columns (Waters, Milford, MA, USA), reconstituted in 100 μL of LCMS methanol, and stored at −80° until UPLC-MS/MS analysis (Moran-Garrido et al. 2025; Watanabe et al. 2024).

### 2.5. Extraction of Free Oxylipins from Serum

Free oxylipins were extracted from 200 μL of serum in 600 μL of premix solution containing 0.002 % BHT, 250 μM EDTA, and 0.01 % acetic acid in methanol/MiliQ water mixture (1:4 v/v) as described in the Supplementary Methods (Moran-Garrido et al. 2025). Samples were vortexed for 5 s, centrifuged at 0 °C for 10 min at 5,000 rcf, and the supernatant was loaded onto 100 mg tC18 Sep-Pak SPE columns (Waters, Milford, MA, USA) to isolate free oxylipins. The extracts were reconstituted in 100 μL of LC-MS/MS grade methanol, and stored in a −80 ° C freezer until UPLC-MS/MS analysis.

### 2.6. GC-EI-MS/MS Analysis

PBDEs, PCBs and PAHs were measured using an Agilent 7890A GC and an Agilent 7000 Triple Quadrupole MS equipped with a Rxi-5sil MS column (15 m × 250 μm × 0.25 μm; Cat # 13620; Restek Corporation, Bellefonte, PA, USA). An aliquot of 2 μL sample was injected by pulsed splitless method, and the split/splitless injector temperature was set at 280 °C. Helium was used as the carrier gas at a flow rate of 1.8 mL/min. The oven temperature program was set initially at 90 °C for 1 min, increased by 50 °C/min to 200 °C and held for 1 min, then increased by 5 °C/min to 260 °C and held for 1 min, finally increased by 50 °C/min to 300 ° C and held for 2 min. The total run time was 20 min.

The MS/MS detector was operated in electronic impact (EI) positive mode at 70 Ev and using optimized multiple reactions monitoring (MRM) conditions (Sup. Table S1). The electron ionization source and transfer line temperature was set at 280 °C and 300 °C, respectively. Accuracy and precision were derived from a 14-point calibration curve and were within the acceptable 80–120 % range.

### 2.7. UPLC-MS/MS Analysis

Oxylipins were measured on an Agilent 1290 LC and Agilent 6460 Triple Quadrupole MS (Agilent Corporation) equipped with an Agilent Eclipse Plus C18 column (ZORBAX RRHD Eclipse Plus 95Å C18, 2.1 x 150 mm, 1.8 μm, 1200 bar, cat No.: 959759–902, Agilent Corporation). Optimized reaction monitoring conditions are shown in Sup. Table S2. The temperature of the auto-sampler was set at 4 °C and the sample injection volume was 10 μL. Mobile phase A contained 0.1 % acetic acid in MilliQ water and Mobile phase B consisted of acetonitrile/methanol (80:15 v/v) containing 0.1 % acetic acid. The mobile phase gradient and pressure program was as follows: 1) 0–2 min, 35 % B, 0.25 mL/min (this was diverted into a waste bottle and not injected into the mass-spec); 2) 2–12 min, 35 to 85 % B, 0.25 mL/min; 3) 12–15 min, 85 % B, 0.25 mL/min; 4) 15.1–17 min, 85 % to 100 % B, 0.4 mL/min; 5) 17.1–19 min, 100 to 35 % B, 0.4 mL/min; and 6) 19–20 min, 35 % B, 0.3 mL/min. The total run time was 20 min.

### 2.8. Gas Chromatography Analysis

Fatty acid methyl esters and total cholesterol were analyzed on a Perkin Elmer Clarus 500 GC-FID system (Perkin Elmer, Shelton, CT, USA) equipped with a DB-FFAP GC column (30 m × 0.25 mm × 0.25 μm; Cat # 122–3232; Agilent Technologies, Santa Clara, CA, USA). An aliquot of 1 μL sample was injected in splitless mode (10:1 split ratio). Helium was used as a carrier gas, at a flow rate of 1.3 mL/min. The temperature of the injector and the detector was maintained at 275 °C and 300 °C, respectively. The oven temperature was programmed as follows: initially the temperature was set at 80 °C for 2 min; increased to 185 °C at the rate of 10 °C/min; increased to 249 °C at the rate of 6 °C/min; and held for 42 min. The total run time was 65 min.

### 2.9. Statistical analysis

When designing the study, we determined detectable geometric mean ratios (with power = 80 %, two-sided alpha = 5 %) when comparing a pre-wildfire control group of size of 30 to a group of size of 150, under varying assumptions about the coefficient of variation. For the smaller comparison group, we can detected geometric mean ratios ranging from 1.44 to 1.92 for a CV ranging from 50 % to 90 %, respectively. For the larger comparison group, the detectable geometric mean ratios decreased to 1.32 to 1.68. The *a priori* power calculation conservatively assumed that we would be considering binary exposure variables, but in the actual analyses, we used continuous exposure variables and assessed associations using correlation and regression methods because we were not able to identify a pre-wildfire control cohort that matched B-SAFE in exposures and other baseline characteristics. We believe that only the actual *a priori* power calculation is appropriate to report, consistent with best practices and use of 95 % CI when reporting the analyses, as these best convey the achieved precision for our final analyses (Tancredi et al. 2021).

Counts and percentages were used to summarize categorical data, and means and standard deviations (mean ± SD) were used to summarize continuous data. All data, including free and total (esterified) oxylipins (pmol/mL), total fatty acids (mg/ml), PBDEs, PCBs and PAHs (ng/mL), and average PM_2.5_ (μg/m^3^) during the peak period were log-transformed (log base 10) to normalize their skewed distributions. An unpaired *t*-test (continuous data) or fisher’s exact test (parametric data) was used to compare postpartum (n = 120) and pregnant (n = 20) subject characteristics and serum chemical, oxylipin and fatty acid concentrations.

A Directed Acyclic Graph (DAG) was constructed to display hypotheses on plausible associations between study variables based on the literature (Sup. Fig. 2) (Greenland et al. 1999), and to determine the specific adjustment variables for regression analyses (Textor et al. 2016). Multiple linear regression was used to test the association between average peak PM_2.5_ (μg/m^3^) (independent variable) to the concentration of each chemical, oxylipin (free and esterified) or fatty acid (dependent variables), adjusting for maternal status (pregnant/postpartum), maternal age at the start of the fire (years), pre-pregnancy BMI calculated using pre-pregnancy weight and height (kg/m^2^), time of sample collection from the start of fire (10–8–2017 was set as the start of fire date) and child’s age at sample collection (weeks). Esterified oxylipin were calculated by subtracting the concentration of free oxylipins from total oxylipin concentrations. Continuous variables were mean centered prior to analysis.

In a sensitivity analysis, we tested for these associations in the postpartum group only (n = 120), adjusting for maternal age at the start of the fire (years), pre-pregnancy BMI, time of sampling from the start of fire and child’s age at sample collection (weeks).

All statistical analyses was performed on SPSS (ver. 28.0.1.1). Associations were considered statistically significant at standard p values of < 0.05. Benjamini-Hochberg False Discovery Rate (FDR) correction was applied at a Q of 5 %.

## 3. Results

### 3.1. Participant Characteristics and PM_2.5_ exposure

All mothers were pregnant when the Tubbs wildfire occurred in October of 2017. By the time blood was taken 7–13 months later, 120 mothers were postpartum and 20 were still pregnant. Table 1 shows baseline subject characteristics at the time of sampling for postpartum (n = 120) and pregnant (n = 20) mothers. Mothers in the cohort were mostly non-Hispanic white (84 %), college educated (99 %), married (98 %), and non-smokers (>99 %). Maternal age, pre-pregnancy BMI and other subject characteristics did not significantly differ between postpartum and pregnant mothers as shown in Table 1.

Out of the 136 participants with available data, 75 evacuated and 61 did not. There were 4 cases where evacuation data were not available (Table 1). Additionally, 63 out of the 140 participants (45 %) returned home after the fires, whereas 77 out of 140 (55 %) permanently relocated. For participants who relocated, PM_2.5_ exposure was assigned based on the residential address corresponding to each of the two peak wildfire exposure days (October 9th and 10th). If a move occurred on one of those dates, PM_2.5_ levels were averaged across the two addresses for that day.

Mean PM_2.5_ peak exposure was 51.6 μg/m^3^ and 49.5 μg/m in mothers who were postpartum and pregnant, respectively, at the time of sample collection (Table 1). In the overall cohort, the 25th percentile PM_2.5_ exposure was 47.77 μg/m^3^, the 75th percentile was 57.24 μg/m^3^, and the 90th percentile was 58.96 μg/m^3^.

### 3.2. Serum PBDEs, PCBs, PAHs, Oxylipins, Fatty Acids, and Diol/Epoxide Ratios in Postpartum and Pregnant Mothers

Sup. Tables S3 and S4 show both lipid-normalized (ng/g of lipid) and non-normalized (ng/mL serum) concentrations of PBDEs, PCBs and PAHs, respectively, in postpartum and pregnant mothers. Lipid normalized values were, for most compounds, higher in postpartum mothers compared pregnant mothers (P < 0.05; Sup. Table S3), but this was due to the significantly lower total lipid values observed in serum of postpartum participants (3.97 ± 2.84 mg/mL) compared to pregnant participants (6.84 ± 3.74 mg/mL; P < 0.001). In the non-lipid normalized data, only PBDE 153 was significantly higher by ~ 3-fold in pregnant compared with postpartum mothers (Sup. Table S4). Non-lipid normalized data were used for the regression analysis (below) to minimize the effects of lipid changes associated with maternal status on PBDEs, PCBs and PAHs (Weldon et al. 2011).

The concentration of 76 measured free, total and esterified oxylipins are shown in Sup. Tables S5, S6 and S7, respectively. Several free oxylipins of the LOX and sEH pathways (free 9,10-DiHOME, 14,15-DiHETrE, 11,12-DiHETrE, 9-HOTrE, 13-HOTrE, 13-HODE, 9-HODE, 13-oxo-ODE, and 9-oxo-ODE) were lower by 118 to 506 % in pregnant compared to postpartum mothers (*P* < 0.05), whereas PGD2 (a COX metabolite) was higher by ~ 2-fold (P < 0.05). Additionally, significant differences were observed in esterified and total oxylipins derived from LOX, CYP450 and sEH, which were generally higher in pregnant mothers compared to postpartum mothers (P < 0.05).

The diol/epoxide ratio, a marker of sEH activation (Stefanovski et al. 2020), is shown in Sup. Table S8. The diol to epoxide ratios of eicosanoids were significantly higher in pregnant mothers compared to postpartum mothers (*P* < 0.05), suggesting broad sEH activation during pregnancy. However, the ratio 12,13-DiHOME/12,13-EpOME from linoleic acid (LA) was lower in pregnant compared to postpartum mothers (*P* < 0.05).

Sup. Table S9 shows fatty acid and cholesterol concentrations. The majority of fatty acids detected were 130 to 274 % higher in pregnant mothers compared to postpartum mothers (P < 0.05). Conversely, eicosapentaenoic acid (EPA) was 74 % lower in pregnant compared to postpartum women (P < 0.05). Total cholesterol was not significantly different between the two groups. Percent fatty acid composition data differed significantly between the groups for several fatty acids as shown in Sup. Table S10.

### 3.3. Associations between PM_2.5_ Exposure and serum lipids

No significant associations were observed between PM_2.5_ exposure and serum fatty acids or cholesterol.

### 3.4. Associations between PM_2.5_ Exposure and oxylipins

An increase in PM_2.5_ exposure was significantly associated with 6 free and esterified oxylipins derived from the LOX pathway (Table 2, Sup. Fig. 3). Specifically, increased PM_2.5_ exposure was associated with lower serum levels of free 9,12,13-TriHOME (β (95 % CI): −0.41 (−0.74, −0.08)), LTB4 (β(95 % CI): −0.85 (−1.68, −0.02)), 9-HOTrE (β (95 % CI): 0.70, (−1.34, −0.07)), 9-oxo-ODE (β (95 % CI): −0.38 (−0.75, −0.1)), and 5-HETE (β (95 % CI): −0.45 (−0.87, −0.03)) (P < 0.05, Table 2). Increased PM_2.5_ exposure was associated with greater serum concentrations of esterified 9-oxo-ODE (β (95 % CI): 0.60 (0.08, 1.12)).

These associations remained significant in a sensitivity analysis which included only the postpartum mothers (n = 120), although additional associations were observed with other LOX-derived oxylipins (Table 3, Sup. Fig. 4). Specifically, an increase in PM_2.5_ exposure was significantly (*P* < 0.05) associated with lower levels of free 20-OH-LTB4 (β (95 % CI): −0.86 (−1.66, −0.05)), and higher levels of esterified 13- oxo-ODE (β (95 % CI): 0.57 (0.12, 1.02)), 5-HETE (β (95 % CI): 0.85, (0.25, 1.45)) and 17-HDoHE (β (95 % CI): 0.59, (0.0008, 1.17)).

None of the associations between PM_2.5_ exposure and oxylipins were significant after FDR correction (Tables 2 and 3).

### 3.5. Associations between PM_2.5_ Exposure and PBDEs, PCBs and PAHs

In both regression analyses incorporating the entire cohort (n = 140) or postpartum mothers only (n = 120), an increase in PM_2.5_ exposure was associated with greater serum levels of the PAH, benzo[k]fluoranthene (Tables 2 and 3). This association was not significant after FDR correction. Additionally, no significant associations were observed between PM_2.5_ exposure and PBDEs, PCBs and other PAHs.

## 4. Discussion

The present findings show that PM_2.5_ from the October 2017 Tubbs fire in California was associated with altered maternal serum free and esterified oxylipin markers of immunity 7 to 13 months after exposure. Specifically, we observed that higher PM_2.5_ exposure was associated with lower serum levels of free LOX-derived oxylipins, and greater esterified LOX-derived oxylipins. We did not observe significant associations between PM_2.5_ exposure and serum chemicals (PBDEs, PCBs and PAHs) and fatty acids, except for a positive association between PM_2.5_ and benzo[k]fluoranthene. These findings should be viewed as prelimenary since none of the significant associations between PM_2.5_ exposure and oxylipins or benzo[k]fluoranthene withstood FDR correction, likely due to the lower than expected sample size.

The inverse association between PM_2.5_ exposure and free LOX-derived eicosanoids (5-HETE and LTB4) and octadecanoids (9-oxo-ODE, 9,12,13-TriHOME and 9-HOTrE), reflects a general reduction in LOX activation 7 to 13 months post-exposure to the Tubbs fire. With the exception of 9-HoTrE which is anti-inflammatory (Pauls et al. 2018), the other LOX metabolites are involved in mediating inflammation, vasodilation or chemotaxis (Chan and Ford-Hutchinson 1985; Dyall et al. 2022). Thus, a dampening of this response many months following exposure to wildfire smoke could reflect adaptive changes in systemic immunity, consistent with a study in non-pregnant juvenile rhesus macaques which showed reduced response of blood mononuclear cells to LPS or flagellin induced inflammation 3 years after exposure to wildfire smoke (Black et al. 2017a).

Interestingly, the inverse association between wildfire PM exposure and LOX-derived oxylipins is opposite to the effects of PM exposure from vehicle exhaust, where an increase in pro-inflammatory cytokines and oxylipins has been reported (Gouveia-Figueira et al. 2017; Gouveia-Figueira et al. 2018; Han et al. 2019; Lam et al. 2020; Li et al. 2015; Wang et al. 2021). Collectively, the evidence points to a dampening of immune responses following PM exposure from wildfires, and an upregulation in immune markers due to PM from other sources (e.g. vehicle exhaust). Differences in lipid-mediated immune responses between PM types are likely due to differences in chemical composition (Koval et al. 2022), and may explain epidemiological observations showing greater risks of respiratory disorders following wildfire PM exposure compared to PM from other sources such as vehicles (Aguilera et al. 2021; Migliaccio et al. 2013).

We observed a positive association between PM_2.5_ exposure and esterified LOX-derived 9-oxo-ODE, opposite to the observed reduction seen in free 9-oxo-ODE with increasing PM_2.5_. Opposite trends in the different lipid pools (free versus esterified) mean that the reduction in free 9-oxo-ODE was likely due to increased esterification. In humans, free oxylipin esterification is catalyzed by acyl-CoA synthetase and acyltransferase enzymes (Golej et al. 2011; Klett et al. 2017). We are not aware of other reports showing an effect of fire smoke PM_2.5_ exposure on these enzymes, although pro-inflammatory oxylipin esterification into phospholipid and neutral lipid pools has been shown to increase in vitro in response to platelets and other inflammatory stimuli (Brezinski and Serhan 1990; Liu et al. 2019). Thus, increased esterification of pro-inflammatory LOX-derived oxylipins in mothers with greater PM_2.5_ exposure, likely reflects an adaptive response to inactivate free oxylipins involved in mediating inflammation. Future studies are needed to examine the relationship between wildfire smoke exposure and enzymes involved in oxylipin esterification as these could serve as modifiable pharmacological targets to mitigate the effects of wildfire PM exposure on LOX-derived oxylipins.

Further stratification of the data by postpartum status showed that PM_2.5_ exposure was associated with greater levels of esterified 9-oxo-ODE, 13-oxo-ODE, 5-HETE and 17-HDoHE of the LOX pathway. Our findings are in agreement with the overall model incorporating both pregnant and postpartum mothers, but further suggest that the response to wildfire PM_2.5_ exposure may be enhanced by postpartum status, as additional LOX-derived esterified oxylipins were associated with greater PM_2.5_ exposure. Differences in maternal oxylipin responses to wildfire smoke during pregnancy and postpartum merit confirmation in future, better powered studies.

Greater PM_2.5_ exposure was associated with higher circulating benzo [k]fluoranthene levels in both the overall cohort (pregnant and postpartum mothers) and postpartum mothers. Benzo[k]fluoranthene is a persistent PAH (Roslund et al. 2018) with an environmental half-life of 1400 days in soil at 20°C (Coover and Sims 1987). It can be generated when tires and roofing material are combusted during fires (Rykała et al. 2022). The volume of distribution and half-life of this compound in humans is not known, making it difficult to establish whether levels of this compound in serum were due to carry over effects from released PM_2.5_ or persistence in soil and housing material once individuals returned to their homes after the fire. The role of benzo[k]fluoranthene on oxylipin-mediated immunity is not known. It would be worthwhile to investigate whether benzo[k]fluoranthene explains some of the serum oxylipin associations observed with fire smoke PM_2.5_ exposure.

The main limitation of this study is the lack of a baseline sample reflecting serum chemical and oxylipin levels prior to PM_2.5_ exposure; this would allow us to establish the extent of change in LOX-derived oxylipins relative to baseline in association with PM_2.5_ exposure. Similarly, it would have been useful to explore temporal changes in oxylipins and benzo[k]fluoranthene shortly after the wildfire to understand when after exposure levels of these compounds change. Another limitation is that PM composition in the soil and in-house dust was not measured to better characterize fire-related persistent exposures that might explain the oxylipin changes. Also, over half the mothers (55 %) relocated to new addresses after the wildfire, making it difficult to understand the potential impact of post-wildfire exposures on oxylipins or benzo[k] fluoranthene. The sample size of 140 mothers was smaller than our target sample size of at least 150 mothers, which is why none of our findings withstood rigorous FDR correction criteria. However, the validity of our findings is strengthened by the fact that we observed significant and reproducible PM_2.5_ associations within multiple but different oxylipin species belonging to the same LOX pathway. Lastly, smoke PM_2.5_ composition was not characterized to establish whether PM_2.5_ from the fire was more enriched with PBDEs, PCBs or their combusted products (e.g. hydroxylated PBDEs and PCBs) compared to background PM_2.5_. As we did not find associations between PM_2.5_ exposure and serum PBDEs and PCBs, knowing smoke PM_2.5_ composition would inform whether exposure to these compounds increased or not during the wildfires. Additionally, measuring serum levels of combusted PBDE and PCB products might have yielded better associations with PM_2.5_, as these compounds have been detected in wildfire ash (Young et al. 2021).

## 5. Conclusions

In summary, our results provide new evidence of immune dysregulation in pregnant and postpartum mothers many months after exposure to an urban wildfire that lasted a few weeks. Our findings point to a dampening of the LOX pathway in both pregnant and lactating mothers 7 to 13 months post-exposure to a wildfire event. Follow-up studies are warranted to understand the implications of these findings to both maternal health and infant development.

## Supplementary Material

1

2

## Figures and Tables

**Table 1:** Mothers’ characteristics in the analysis from B-SAFE cohort (N = 140).

Characteristic	B-SAFE Postpartum^a^ N = 120	B-SAFE Pregnant^b^ N =20	p value^c^
**Demographics**			
Mother’s age at sampling (yrs)	33.7 ± 3.7 (n = 4 missing)	32.1 ± 3.9	0.105
Mother’s age at conception (yrs)	32.5 ± 3.7 (n = 4 missing)	31.5 ± 3.8	0.287
Baby’s age at sampling (weeks)	26.9 ± 11.7 (n = 1 missing)	NA	NA
Relationship status (married or living as married)	105 (97 %) (n = 12 missing)	19 (100 %) (n = 1 missing)	
**Highest education**			
Below 4 years degree (high school diploma/GED, college)	19 (16 %)	9 (45 %)	
4 years degree (bachelor)	59 (49 %)	5 (25 %)	
Graduate degree	42 (35 %)	6 (30 %)	
**Race/Ethnicity**			
White (non-Hispanic)	95 (82 %) (n = 7 missing)	14 (78 %) (n = 3 missing)	
Non-White (non-Hispanic)	10 (9 %) (n = 7 missing)	0 (0 %) (n = 3 missing)	
Hispanic (any race)	11 (9 %) (n = 4 missing)	4 (22 %) (n = 2 missing)	0.120
**Pregnancy data**			
Prepregnant BMI (kg/m^b)^	25.8 ± 6.8	27.4 ± 8.4 (n = 1 missing)	0.426
Prepregnant weight (pounds; lbs)	156.7 ± 42.2	163.5 ± 45.5 (n = 1 missing)	0.550
Delivery weight (pounds; lbs)	187.0 ± 42.5 (n = 3 missing)	193.1 ± 43.3 (n = 1 missing)	0.572
Pregnant weight gain (pounds; lbs)	29.9 ± 13.5 (n = 3 missing)	29.6 ± 16.0 (n = 1 missing)	0.943
Was the child exclusively breastfed? (Yes (%))	68 (57 %) (n = 3 missing)	NA	
**Smoking status**			
Self (Yes (%))	1 (0.8 %)	0 (0 %) (n = 1 missing)	>0.999
**Fire exposure**			
Sample collection time-point since the start of the Tubbs fire (months)^d^	10.6 ± 1.2	8.9 ± 1.0	< 0.001
Pregnant during fire	118 (98 %)	8 (40 %)	< 0.001
Average peak PM (μg/m^c^)	50.1 ± 9.6	43.6 ± 13.6	0.026
Number and percentage of individuals who evacuated their homes during the wildfire^e^	64 (55 %) (n = 3 missing)	11 (58 %) (n = 1 missing)	<0.001

NA: not applicable. No data available.

a Reflects the number of postpartum mothers at the time of sample collection.

b Reflects the number of pregnant mothers at the time of sample collection.

c T-test for continuous variables or fisher’s exact test for categorical variables comparing postpartum mothers to pregnant mothers.

d Number of months from the 2017 Tubbs Fire that the sample was collected (October 8th, 2017 was set as the start of fire time-point).

e Evacuation status was determined by identifying and counting mothers who evacuated their homes during the Tubbs fire.

**Table 2:** Association between PM_2.5_ exposure and oxylipins (free and esterified) and PAHs in pregnant (n = 20) and postpartum (n = 120) women, 7 to 13 months post-exposure to smoke PM_2.5_ from the Tubbs fire (n = 140 total).

Groups	Compounds	β (95 % CI)	P-value	Benjamini-Hochberg Critical Value (5 %)	Precursor lipid	Related Enzyme
Free oxylipins	9,12,13-TriHOME	−0.41 (−0.736, −0.085)	0.014	0.0004	LA	LOX
	LTB4	−0.85 (−1.680, −0.020)	0.045	0.0020	ARA	LOX
	9-HOTrE	−0.70 ( −1.340, −0.068)	0.030	0.0008	ALA	LOX
	9-oxo-ODE	−0.38 ( −0.752, −0.009)	0.045	0.0016	LA	LOX
	5-HETE	−0.45 ( −0.873, −0.032)	0.035	0.0012	ARA	LOX
PAHs	Benzo[k]fluoranthene (adjusted by volume)	0.87 (0.039, 1.690)	0.040	0.0005	NA	NA
Esterified oxylipins	9-oxo-ODE0	60 (0.077, 1.120)	0.025	0.0019	LA	LOX

* NA: not applicable. No data available.

Multiple linear regression was used to test the association between average peak PM2.5 (μg/m3) (independent variable) to the concentration of each chemical, oxylipin or fatty acid (dependent variables, adjusting for maternal status (pregnant/postpartum), maternal age at the start of the fire (years), pre-pregnancy BMI calculated using pre-pregnancy weight and height (kg/m2), time of sampling from the start of fire and child’s age at sample collection (weeks), as a surrogate for breastfeeding duration. Continuous variables were mean centered. The table reports significant associations at a p-value of <0.05

**Table 3:** Association between PM_2.5_ exposure and oxylipins (free and esterified) and PAHs in postpartum (n = 120) women, 7 to 13 months post-exposure to smoke PM_2.5_ from the Tubbs fire.

Groups	Compounds	β (95 % CI)	P-value	Benjamini-Hochberg Critical Value (5 %)	Precursor lipid	Related Enzyme
Free oxylipin	20-OH-LTB4	−0.86 (−1.662, −0.049)	0.038	0.0025	ARA	LOX
	9,12,13-TriHOME	−0.53 (−0.905, −0.152)	0.006	0.0004	LA	LOX
	LTB4	−1.03 (−2.004, −0.066)	0.037	0.0020	ARA	LOX
	9-HOTrE	−0.96 (−1.728, −0.199)	0.014	0.0008	ALA	LOX
	9-oxo-ODE	−0.48 (−0.935, −0.035)	0.035	0.0016	LA	LOX
Total oxylipin	9-oxo-ODE	0.3 (0.029, 0.576)	0.030	0.0012	LA	LOX
PAHs	Benzo[k]fluoranthene (adjusted by volume)	1.05 (0.016, 2.086)	0.047	0.0005	NA	NA
Esterified oxylipin	13-oxo-ODE	0.57 (0.121, 1.023)	0.014	0.0032	LA	LOX
	9-oxo-ODE	0.95 (0.313, 1.594)	0.004	0.0013	LA	LOX
	5-HETE	0.85 (0.248, 1.446)	0.006	0.0019	ARA	LOX
	17-HDoHE	0.59 (0.001, 1.172)	0.050	0.0044	DHA	LOX

* NA: not applicable. No data available.

In a sensitivity analysis, multiple linear regression was used to test the association between average peak PM2.5 (μg/m3) (independent variable) to the concentration of each chemical, oxylipin or fatty acid (dependent variables) in postpartum women (n = 120), adjusting for maternal age at the start of the fire (years), pre-pregnancy BMI calculated using pre-pregnancy weight and height (kg/m2), time of sampling from the start of fire and child’s age at sample collection (weeks), as a surrogate for breastfeeding duration. Continuous variables were mean centered. The table reports significant associations at a p-value of <0.05.

## Data Availability

Data will be made available on request.

## Appendix A. Supplementary data

Supplementary data to this article can be found online at https://doi.org/10.1016/j.envint.2025.109468.

## Abbreviations:

PAH: polycyclic aromatic hydrocarbon
PBDE: polybrominated diphenyl ether
PCB: polychlorinated biphenyl
PM: particulate matter
GC-EI-MS/MS: gas chromatography-electron ionization-tandem mass spectrometry
SPE: solid phase extraction
NIST: National Institute of Standards and Technology
PGB2: Prostaglandin B2
AA: arachidonic acid
EPA: eicosapentaenoic acid
11,12-DiHETE: 11,12-dihydroxy-5Z,8Z,14Z,17Z-eicosatetraenoic acid
19,20-EpDPE: 19(20)-epoxydocosapentaenoic acid
7,8-EpDPE: 7(8)-epoxydocosapentaenoic acid
13-oxo-ODE: 13-oxo-octadecadienoic acid
15-oxo-ETE: 15-oxo-eicosatetraenoic acid
17-HDoHE: 17- hydroxydocosahexaenoic acid
20-OH-LTB4: 20-OH-Leukotriene B4
5-HEPE: 5-hydroxyeicosapentaenoic acid
5-HETE: 5-hydroxyeicosatetraenoic acid
9,12,13-TriHOME: 9,12,13-trihydroxyoctadecamonoenoic acid
9-HETE: 9-hydroxyeicosatetraenoic acid
9-HOTrE: 9- hydroxyoctadecatrienoic acid
9-oxo-ODE: 9-oxo-octadecadienoic acid
LTE4: Leukotriene E4
11,12-DiHETrE: 11,12-dihydroxyeicosatrienoic acid
17,18-DiHETE: 17,18-dihydroxyeicosatetraenoic acid
8,9-DiHETE: 8,9-dihydroxy-5Z,11Z,14Z,17Z-eicosatetraenoic acid
8,9-DiHETrE: 8,9-dihydroxyeicosatrienoic acid
19,20-DiHDPA: 19,20-dihydroxy-4Z,7Z,10Z,13Z,16Z-docosapentaenoic acid
COX: cyclooxygenase
LOX: lipoxygenase
CYP450: cytochrome P450
she: soluble epoxide hydrolase
