# Supplementary material for: Gestational exposure to particulate matter from urban wildfires is associated with changes in circulating oxylipins but not flame retardants 7 to 13 months post-exposure
Source: Environ Int. Author manuscript; Available in PMC 2026 May 26. (PMC13201207; doi:10.1016/j.envint.2025.109468)
Supplement: 1 [file NIHMS2174401-supplement-1.docx]

Supplementary Material

**Supplementary Table 1.** Acquisition method for PBDEs, PCBs, PAHs, and their labeled internal standards under multiple reaction monitoring (MRM) mode.

| **Compound Name** | **ISTD** | **Precursor ion (m/z)** | **Product ion (m/z)** | **RT (min)** | **Left Delta RT (min)** | **Right Delta RT (min)** | **Dwell time (ms)** | **CE (V)** |
| --- | --- | --- | --- | --- | --- | --- | --- | --- |
| PBDE 100 | 13C-PBDE 118 | 563.8 | 403.8 | 11.32 | 0.50 | 0.50 | 19.5 | 25 |
| PBDE 100 | 13C-PBDE 118 | 405.8 | 299 | 11.32 | 0.50 | 0.50 | 19.5 | 35 |
| PBDE 100 | 13C-PBDE 118 | 405.8 | 296.9 | 11.32 | 0.50 | 0.50 | 19.5 | 40 |
| PBDE 100 | 13C-PBDE 118 | 405.7 | 296.9 | 11.32 | 0.50 | 0.50 | 19.5 | 40 |
| PBDE 136 | 13C-PBDE 118 | 643.6 | 483.6 | 15.08 | 0.50 | 0.50 | 24.1 | 35 |
| PBDE 136 | 13C-PBDE 118 | 483.7 | 374.8 | 15.08 | 0.50 | 0.50 | 24.1 | 40 |
| PBDE 136 | 13C-PBDE 118 | 483.7 | 324 | 15.08 | 0.50 | 0.50 | 24.1 | 45 |
| PBDE 136 | 13C-PBDE 118 | 483.6 | 324 | 15.08 | 0.50 | 0.50 | 24.1 | 40 |
| PBDE 153 | 13C-PBDE 118 | 643.5 | 483.6 | 15.24 | 0.50 | 0.50 | 33.6 | 35 |
| PBDE 153 | 13C-PBDE 118 | 483.7 | 324 | 15.24 | 0.50 | 0.50 | 33.6 | 45 |
| PBDE 153 | 13C-PBDE 118 | 483.6 | 376.8 | 15.24 | 0.50 | 0.50 | 33.6 | 40 |
| PBDE 153 | 13C-PBDE 118 | 483.6 | 323.9 | 15.24 | 0.50 | 0.50 | 33.6 | 45 |
| PBDE 154 | 13C-PBDE 118 | 643.4 | 483.6 | 14.10 | 0.50 | 0.50 | 33.6 | 25 |
| PBDE 154 | 13C-PBDE 118 | 484 | 377 | 14.10 | 0.50 | 0.50 | 33.6 | 35 |
| PBDE 154 | 13C-PBDE 118 | 484 | 374.9 | 14.10 | 0.50 | 0.50 | 33.6 | 40 |
| PBDE 154 | 13C-PBDE 118 | 483.7 | 374.8 | 14.10 | 0.50 | 0.50 | 33.6 | 40 |
| PBDE 183 | 13C-PBDE 118 | 721.5 | 561.6 | 17.97 | 0.50 | 0.50 | 40.6 | 25 |
| PBDE 183 | 13C-PBDE 118 | 721.4 | 561.6 | 17.97 | 0.50 | 0.50 | 40.6 | 25 |
| PBDE 183 | 13C-PBDE 118 | 561.7 | 454.6 | 17.97 | 0.50 | 0.50 | 40.6 | 35 |
| PBDE 183 | 13C-PBDE 118 | 561.6 | 454.6 | 17.97 | 0.50 | 0.50 | 40.6 | 35 |
| PBDE 47 | 13C-PBDE 118 | 485.7 | 325.7 | 9.07 | 0.50 | 0.50 | 9.3 | 20 |
| PBDE 47 | 13C-PBDE 118 | 485.6 | 325.7 | 9.07 | 0.50 | 0.50 | 9.3 | 25 |
| PBDE 47 | 13C-PBDE 118 | 326 | 219 | 9.07 | 0.50 | 0.50 | 9.3 | 35 |
| PBDE 47 | 13C-PBDE 118 | 325.7 | 217 | 9.07 | 0.50 | 0.50 | 9.3 | 30 |
| PBDE 49 | 13C-PBDE 118 | 485.7 | 325.9 | 8.63 | 0.50 | 0.50 | 7.3 | 20 |
| PBDE 49 | 13C-PBDE 118 | 485.6 | 325.7 | 8.63 | 0.50 | 0.50 | 7.3 | 25 |
| PBDE 49 | 13C-PBDE 118 | 325.9 | 218.9 | 8.63 | 0.50 | 0.50 | 7.3 | 35 |
| PBDE 49 | 13C-PBDE 118 | 325.8 | 217 | 8.63 | 0.50 | 0.50 | 7.3 | 30 |
| PBDE 52 | 13C-PBDE 118 | 485.6 | 325.9 | 8.22 | 0.50 | 0.50 | 8.8 | 20 |
| PBDE 52 | 13C-PBDE 118 | 485.6 | 325.7 | 8.22 | 0.50 | 0.50 | 8.8 | 25 |
| PBDE 52 | 13C-PBDE 118 | 325.9 | 218.9 | 8.22 | 0.50 | 0.50 | 8.8 | 30 |
| PBDE 52 | 13C-PBDE 118 | 485.7 | 325.9 | 8.63 | 0.50 | 0.50 | 7.3 | 20 |
| PBDE 66 | 13C-PBDE 118 | 485.6 | 325.7 | 9.52 | 0.50 | 0.50 | 12 | 25 |
| PBDE 66 | 13C-PBDE 118 | 483.6 | 323.8 | 9.52 | 0.50 | 0.50 | 12 | 20 |
| PBDE 66 | 13C-PBDE 118 | 325.9 | 217 | 9.52 | 0.50 | 0.50 | 12 | 30 |
| PBDE 66 | 13C-PBDE 118 | 325.7 | 217 | 9.52 | 0.50 | 0.50 | 12 | 30 |
| PBDE 85 | 13C-PBDE 118 | 563.5 | 403.7 | 12.12 | 0.50 | 0.50 | 16.9 | 25 |
| PBDE 85 | 13C-PBDE 118 | 403.9 | 296.9 | 13.40 | 0.50 | 0.50 | 33.1 | 30 |
| PBDE 85 | 13C-PBDE 118 | 403.9 | 294.9 | 13.40 | 0.50 | 0.50 | 33.1 | 35 |
| PBDE 85 | 13C-PBDE 118 | 403.9 | 244 | 13.40 | 0.50 | 0.50 | 33.1 | 40 |
| PBDE 95 | 13C-PBDE 118 | 563.8 | 403.8 | 11.10 | 0.50 | 0.50 | 27 | 25 |
| PBDE 95 | 13C-PBDE 118 | 405.8 | 299 | 11.10 | 0.50 | 0.50 | 27 | 35 |
| PBDE 95 | 13C-PBDE 118 | 405.8 | 296.9 | 11.10 | 0.50 | 0.50 | 27 | 40 |
| PBDE 95 | 13C-PBDE 118 | 405.7 | 296.9 | 11.10 | 0.50 | 0.50 | 27 | 40 |
| PBDE 99 | 13C-PBDE 118 | 565.3 | 405.8 | 12.12 | 0.50 | 0.50 | 16.9 | 25 |
| PBDE 99 | 13C-PBDE 118 | 403.9 | 296.9 | 12.12 | 0.50 | 0.50 | 16.9 | 30 |
| PBDE 99 | 13C-PBDE 118 | 403.9 | 294.9 | 12.12 | 0.50 | 0.50 | 16.9 | 35 |
| PBDE 99 | 13C-PBDE 118 | 403.9 | 243.9 | 12.12 | 0.50 | 0.50 | 16.9 | 40 |
| PBDE 28 | 13C-PBDE 28 | 407.6 | 248 | 6.54 | 0.50 | 0.50 | 7.1 | 25 |
| PBDE 28 | 13C-PBDE 28 | 407.6 | 247.7 | 6.54 | 0.50 | 0.50 | 7.1 | 20 |
| PBDE 28 | 13C-PBDE 28 | 245.7 | 138.8 | 6.54 | 0.50 | 0.50 | 7.1 | 30 |
| PCB 11 | 13C-PCB 11 | 222 | 152 | 3.73 | 0.50 | 0.50 | 16 | 25 |
| PCB 11 | 13C-PCB 11 | 152 | 151.1 | 3.73 | 0.50 | 0.50 | 16 | 25 |
| PCB 28 | 13C-PCB 11 | 255.8 | 186 | 4.13 | 0.50 | 0.50 | 24.6 | 35 |
| PCB 28 | 13C-PCB 11 | 255.8 | 151 | 4.13 | 0.50 | 0.50 | 24.6 | 45 |
| PCB 52 | 13C-PCB 11 | 291.8 | 222 | 4.33 | 0.50 | 0.50 | 24.8 | 30 |
| PCB 52 | 13C-PCB 11 | 291.8 | 220 | 4.33 | 0.50 | 0.50 | 24.8 | 30 |
| PCB 66 | 13C-PCB 11 | 291.8 | 222 | 5.05 | 0.50 | 0.50 | 15.3 | 30 |
| PCB 66 | 13C-PCB 11 | 291.8 | 220 | 5.05 | 0.50 | 0.50 | 15.3 | 30 |
| PCB 101 | 13C-PCB 97 | 327.6 | 255.7 | 5.44 | 0.50 | 0.50 | 7.8 | 30 |
| PCB 101 | 13C-PCB 97 | 325.6 | 256.1 | 5.44 | 0.50 | 0.50 | 7.8 | 25 |
| PCB 101 | 13C-PCB 97 | 325.6 | 255.9 | 5.44 | 0.50 | 0.50 | 7.8 | 35 |
| PCB 101 | 13C-PCB 97 | 253.7 | 183.7 | 5.44 | 0.50 | 0.50 | 7.8 | 30 |
| PCB 118 | 13C-PCB 97 | 325.6 | 256 | 6.46 | 0.50 | 0.50 | 7 | 30 |
| PCB 118 | 13C-PCB 97 | 325.6 | 254 | 6.46 | 0.50 | 0.50 | 7 | 25 |
| PCB 118 | 13C-PCB 97 | 323.5 | 253.9 | 6.46 | 0.50 | 0.50 | 7 | 30 |
| PCB 126 | 13C-PCB 97 | 327.8 | 255.9 | 7.58 | 0.50 | 0.50 | 11 | 30 |
| PCB 126 | 13C-PCB 97 | 253.9 | 184 | 7.58 | 0.50 | 0.50 | 11 | 35 |
| PCB 131 | 13C-PCB 97 | 359.6 | 290 | 6.72 | 0.50 | 0.50 | 7.6 | 20 |
| PCB 131 | 13C-PCB 97 | 289.6 | 219.9 | 6.72 | 0.50 | 0.50 | 7.6 | 30 |
| PCB 131 | 13C-PCB 97 | 289.6 | 217.9 | 6.72 | 0.50 | 0.50 | 7.6 | 35 |
| PCB 131 | 13C-PCB 97 | 287.6 | 217.7 | 6.72 | 0.50 | 0.50 | 7.6 | 35 |
| PCB 132 | 13C-PCB 97 | 359.6 | 290 | 6.88 | 0.50 | 0.50 | 8.7 | 20 |
| PCB 132 | 13C-PCB 97 | 289.6 | 220 | 6.88 | 0.50 | 0.50 | 8.7 | 30 |
| PCB 132 | 13C-PCB 97 | 289.6 | 217.7 | 6.88 | 0.50 | 0.50 | 8.7 | 35 |
| PCB 135 | 13C-PCB 97 | 359.6 | 290 | 6.25 | 0.50 | 0.50 | 7.6 | 25 |
| PCB 135 | 13C-PCB 97 | 289.6 | 220 | 6.25 | 0.50 | 0.50 | 7.6 | 30 |
| PCB 135 | 13C-PCB 97 | 289.6 | 218 | 6.25 | 0.50 | 0.50 | 7.6 | 35 |
| PCB 136 | 13C-PCB 97 | 359.6 | 290 | 5.93 | 0.50 | 0.50 | 7.7 | 25 |
| PCB 136 | 13C-PCB 97 | 289.6 | 219.9 | 5.93 | 0.50 | 0.50 | 7.7 | 30 |
| PCB 136 | 13C-PCB 97 | 289.6 | 218 | 5.93 | 0.50 | 0.50 | 7.7 | 35 |
| PCB 138 | 13C-PCB 97 | 360 | 290 | 7.40 | 0.50 | 0.50 | 10.5 | 40 |
| PCB 138 | 13C-PCB 97 | 359.6 | 290 | 7.40 | 0.50 | 0.50 | 10.5 | 30 |
| PCB 138 | 13C-PCB 97 | 291.9 | 220 | 7.40 | 0.50 | 0.50 | 10.5 | 35 |
| PCB 138 | 13C-PCB 97 | 289.9 | 220 | 7.40 | 0.50 | 0.50 | 10.5 | 40 |
| PCB 149 | 13C-PCB 97 | 359.6 | 290 | 6.38 | 0.50 | 0.50 | 7.5 | 20 |
| PCB 149 | 13C-PCB 97 | 289.6 | 219.9 | 6.38 | 0.50 | 0.50 | 7.5 | 30 |
| PCB 149 | 13C-PCB 97 | 289.6 | 217.7 | 6.38 | 0.50 | 0.50 | 7.5 | 35 |
| PCB 153 | 13C-PCB 97 | 359.6 | 290 | 6.87 | 0.50 | 0.50 | 8.2 | 20 |
| PCB 153 | 13C-PCB 97 | 287.6 | 218 | 6.87 | 0.50 | 0.50 | 8.2 | 40 |
| PCB 153 | 13C-PCB 97 | 287.6 | 217.7 | 6.87 | 0.50 | 0.50 | 8.2 | 35 |
| PCB 174 | 13C-PCB 97 | 393.7 | 323.9 | 8.24 | 0.50 | 0.50 | 8 | 35 |
| PCB 174 | 13C-PCB 97 | 393.5 | 323.6 | 8.24 | 0.50 | 0.50 | 8 | 30 |
| PCB 174 | 13C-PCB 97 | 323.9 | 253.9 | 8.24 | 0.50 | 0.50 | 8 | 30 |
| PCB 175 | 13C-PCB 97 | 393.7 | 323.9 | 7.66 | 0.50 | 0.50 | 10.6 | 35 |
| PCB 175 | 13C-PCB 97 | 393.5 | 323.6 | 7.66 | 0.50 | 0.50 | 10.6 | 20 |
| PCB 176 | 13C-PCB 97 | 393.8 | 323.9 | 7.25 | 0.50 | 0.50 | 10 | 35 |
| PCB 176 | 13C-PCB 97 | 393.5 | 323.6 | 7.25 | 0.50 | 0.50 | 10 | 20 |
| PCB 180 | 13C-PCB 97 | 395.9 | 323.8 | 8.95 | 0.50 | 0.50 | 8 | 30 |
| PCB 180 | 13C-PCB 97 | 393.5 | 323.6 | 8.95 | 0.50 | 0.50 | 8 | 30 |
| PCB 180 | 13C-PCB 97 | 325.6 | 256 | 8.95 | 0.50 | 0.50 | 8 | 40 |
| PCB 196 | 13C-PCB 97 | 429.5 | 359.8 | 9.99 | 0.50 | 0.50 | 30.8 | 30 |
| PCB 196 | 13C-PCB 97 | 427.6 | 357.9 | 9.99 | 0.50 | 0.50 | 30.8 | 35 |
| PCB 196 | 13C-PCB 97 | 357.9 | 287.9 | 9.99 | 0.50 | 0.50 | 30.8 | 45 |
| PCB 197 | 13C-PCB 97 | 429.9 | 359.9 | 8.69 | 0.50 | 0.50 | 7.5 | 30 |
| PCB 197 | 13C-PCB 97 | 429.8 | 359.9 | 8.69 | 0.50 | 0.50 | 7.5 | 30 |
| PCB 197 | 13C-PCB 97 | 179.2 | 107.6 | 8.69 | 0.50 | 0.50 | 7.5 | 30 |
| PCB 197 | 13C-PCB 97 | 179.1 | 143.9 | 8.69 | 0.50 | 0.50 | 7.5 | 25 |
| PCB 202 | 13C-PCB 97 | 429.9 | 359.9 | 8.50 | 0.50 | 0.50 | 7.5 | 30 |
| PCB 202 | 13C-PCB 97 | 429.8 | 359.9 | 8.50 | 0.50 | 0.50 | 7.5 | 30 |
| PCB 202 | 13C-PCB 97 | 179.1 | 143.9 | 8.50 | 0.50 | 0.50 | 7.5 | 25 |
| PCB 77 | 13C-PCB 97 | 291.8 | 222 | 6.02 | 0.50 | 0.50 | 7.6 | 30 |
| PCB 77 | 13C-PCB 97 | 291.8 | 220 | 6.02 | 0.50 | 0.50 | 7.6 | 30 |
| PCB 77 | 13C-PCB 97 | 289.6 | 219.9 | 6.02 | 0.50 | 0.50 | 7.6 | 30 |
| PCB 84 | 13C-PCB 97 | 327.6 | 255.7 | 5.39 | 0.50 | 0.50 | 8.3 | 30 |
| PCB 84 | 13C-PCB 97 | 325.6 | 255.9 | 5.39 | 0.50 | 0.50 | 8.3 | 25 |
| PCB 84 | 13C-PCB 97 | 253.7 | 183.7 | 5.39 | 0.50 | 0.50 | 8.3 | 30 |
| PCB 91 | 13C-PCB 97 | 327.6 | 255.7 | 5.19 | 0.50 | 0.50 | 10.7 | 30 |
| PCB 91 | 13C-PCB 97 | 325.7 | 255.9 | 5.19 | 0.50 | 0.50 | 10.7 | 35 |
| PCB 91 | 13C-PCB 97 | 253.7 | 183.7 | 5.19 | 0.50 | 0.50 | 10.7 | 30 |
| PCB 95 | 13C-PCB 97 | 327.6 | 255.7 | 5.11 | 0.50 | 0.50 | 13.2 | 30 |
| PCB 95 | 13C-PCB 97 | 325.6 | 255.9 | 5.11 | 0.50 | 0.50 | 13.2 | 25 |
| PCB 95 | 13C-PCB 97 | 253.7 | 183.7 | 5.11 | 0.50 | 0.50 | 13.2 | 30 |
| Acenaphthylene | D12 Chrysene | 152 | 150.1 | 2.77 | 0.70 | 0.50 | 39.8 | 35 |
| Acenaphthylene | D12 Chrysene | 150 | 149.9 | 2.77 | 0.70 | 0.50 | 39.8 | 35 |
| Acenaphthene | D12 Chrysene | 153.9 | 151.9 | 2.86 | 0.50 | 0.50 | 25.7 | 35 |
| Acenaphthene | D12 Chrysene | 153 | 77 | 2.86 | 0.50 | 0.50 | 25.7 | 45 |
| Anthracene | D12 Chrysene | 177.9 | 176.1 | 3.75 | 0.50 | 0.50 | 16.3 | 40 |
| Anthracene | D12 Chrysene | 177.9 | 152 | 3.75 | 0.50 | 0.50 | 16.3 | 25 |
| Benzo[ghi]perylene | D12 Chrysene | 275.9 | 274.1 | 16.99 | 0.50 | 0.50 | 51.9 | 45 |
| Benz[a]anthracene | D12 Chrysene | 228.1 | 228.1 | 8.25 | 0.50 | 0.50 | 7.9 | 35 |
| Benz[a]anthracene | D12 Chrysene | 228.1 | 226.1 | 8.25 | 0.50 | 0.50 | 7.9 | 35 |
| Benzo[a]pyrene | D12 Chrysene | 251.9 | 250.1 | 12.65 | 0.50 | 0.50 | 18.3 | 40 |
| Benzo[a]pyrene | D12 Chrysene | 249.9 | 248.1 | 12.65 | 0.50 | 0.50 | 18.3 | 40 |
| Benzo[b]fluoranthene | D12 Chrysene | 251.9 | 250.1 | 11.65 | 0.50 | 0.50 | 18.7 | 45 |
| Benzo[b]fluoranthene | D12 Chrysene | 249.9 | 248.1 | 11.65 | 0.50 | 0.50 | 18.7 | 40 |
| Benzo[ghi]perylene | D12 Chrysene | 138.2 | 136.1 | 16.99 | 0.50 | 0.50 | 51.9 | 35 |
| Benzo[k]fluoranthene | D12 Chrysene | 251.8 | 250.1 | 11.75 | 0.50 | 0.50 | 18.6 | 40 |
| Benzo[k]fluoranthene | D12 Chrysene | 249.9 | 248.1 | 11.75 | 0.50 | 0.50 | 18.6 | 40 |
| Chrysene | D12 Chrysene | 227.9 | 227.1 | 8.43 | 0.50 | 0.50 | 7.6 | 35 |
| Chrysene | D12 Chrysene | 227.9 | 226.1 | 8.43 | 0.50 | 0.50 | 7.6 | 35 |
| Chrysene | D12 Chrysene | 226.1 | 202.1 | 8.43 | 0.50 | 0.50 | 7.6 | 40 |
| Dibenz[a,h]anthracene | D12 Chrysene | 277.8 | 276.1 | 16.47 | 0.50 | 0.50 | 38.6 | 40 |
| Dibenzo-ah-anthracene | D12 Chrysene | 139.1 | 112.1 | 16.47 | 0.50 | 0.50 | 38.6 | 30 |
| Fluoranthene | D12 Chrysene | 201.9 | 200.1 | 5.04 | 0.50 | 0.50 | 15.3 | 45 |
| Fluoranthene | D12 Chrysene | 201.9 | 201.1 | 5.40 | 0.50 | 0.50 | 8.3 | 25 |
| Fluorine | D12 Chrysene | 165.9 | 165.1 | 3.14 | 0.50 | 0.50 | 19.5 | 25 |
| Fluorine | D12 Chrysene | 165.9 | 164.1 | 3.14 | 0.50 | 0.50 | 19.5 | 40 |
| Indeno[1,2,3-cd]pyrene | D12 Chrysene | 275.8 | 274.1 | 16.24 | 0.50 | 0.50 | 31.4 | 45 |
| Indeno[1,2,3-cd]pyrene | D12 Chrysene | 138.1 | 136.9 | 16.24 | 0.50 | 0.50 | 31.4 | 25 |
| Indeno[1,2,3-cd]pyrene | D12 Chrysene | 136.9 | 124.1 | 16.24 | 0.50 | 0.50 | 31.4 | 30 |
| Phenanthrene | D12 Chrysene | 177.9 | 152 | 3.75 | 0.50 | 0.50 | 16.3 | 25 |
| Phenanthrene | D12 Chrysene | 177.9 | 176.2 | 3.83 | 0.50 | 0.50 | 18.9 | 40 |
| Phenanthrene | D12 Chrysene | 175.9 | 176 | 3.83 | 0.50 | 0.50 | 18.9 | 25 |
| Pyrene | D12 Chrysene | 202.1 | 201.1 | 5.40 | 0.50 | 0.50 | 8.3 | 30 |
| Pyrene | D12 Chrysene | 201.1 | 200.1 | 5.40 | 0.50 | 0.50 | 8.3 | 45 |
| 13C PBDE 118 | X | 418.1 | 307.8 | 12.77 | 0.50 | 0.50 | 19.3 | 35 |
| 13C PBDE 118 | X | 418.1 | 257.8 | 12.77 | 0.50 | 0.50 | 19.3 | 35 |
| 13C PBDE 118 | X | 417.7 | 307.9 | 12.77 | 0.50 | 0.50 | 19.3 | 35 |
| 13C PBDE 118 | X | 417.7 | 257.9 | 12.77 | 0.50 | 0.50 | 19.3 | 35 |
| 13C PBDE 118 | X | 417.6 | 307.9 | 12.77 | 0.50 | 0.50 | 19.3 | 30 |
| 13C PBDE 118 | X | 417.6 | 257.9 | 12.77 | 0.50 | 0.50 | 19.3 | 30 |
| 13C PBDE 28 | X | 417.9 | 260 | 6.54 | 0.50 | 0.50 | 6.8 | 20 |
| 13C PBDE 28 | X | 417.9 | 258 | 6.54 | 0.50 | 0.50 | 6.8 | 25 |
| 13C PBDE 28 | X | 417.7 | 260 | 6.54 | 0.50 | 0.50 | 6.8 | 20 |
| 13C PBDE 28 | X | 417.7 | 258 | 6.54 | 0.50 | 0.50 | 6.8 | 25 |
| 13C PCB 11 | X | 236 | 164.1 | 3.70 | 0.50 | 0.50 | 17 | 30 |
| 13C PCB 11 | X | 234 | 164.1 | 3.70 | 0.50 | 0.50 | 17 | 25 |
| 13C PCB 97 | X | 337.5 | 268 | 5.73 | 0.50 | 0.50 | 7.9 | 25 |
| 13C PCB 97 | X | 265.8 | 231.1 | 5.73 | 0.50 | 0.50 | 7.9 | 30 |
| 13C PCB 97 | X | 265.8 | 196.1 | 5.73 | 0.50 | 0.50 | 7.9 | 35 |
| 13C PCB 97 | X | 265.7 | 195.8 | 5.73 | 0.50 | 0.50 | 7.9 | 35 |
| D12 Chrysene | X | 240.2 | 236.2 | 8.40 | 0.50 | 0.50 | 7.7 | 35 |
| D12 Chrysene | X | 239.9 | 236.1 | 8.40 | 0.50 | 0.50 | 7.7 | 35 |
| D12 Chrysene | X | 235.8 | 232.1 | 8.40 | 0.50 | 0.50 | 7.7 | 35 |
| Mirex | X | 273.9 | 238.8 | 9.60 | 0.50 | 0.50 | 14.7 | 20 |
| Mirex | X | 271.9 | 236.9 | 9.60 | 0.50 | 0.50 | 14.7 | 20 |
| Mirex | X | 236.9 | 142.9 | 9.60 | 0.50 | 0.50 | 14.7 | 30 |
| Mirex | X | 236.9 | 118.9 | 9.60 | 0.50 | 0.50 | 14.7 | 30 |

ISTD: internal standard

RT: retention time

CE: collision energy to generate product ions

**Supplementary Table 2.** Acquisition method of oxylipins and deuterated internal standards in multiple reaction monitoring (MRM) mode.

| Compound Name | Precursor ion (m/z) | Product ion (m/z) | RT (min) | Delta RT (min) | CE (V) |
| --- | --- | --- | --- | --- | --- |
| 11(12)-EpETE | 317.2 | 167.2 | 12.9 | 1.4 | 10 |
| 11(12)-EpETrE | 319.2 | 167.2 | 13.93 | 1.2 | 10 |
| 11,12-DiHErTE | 337.2 | 167.1 | 10.78 | 1.3 | 12 |
| 11,12DiHETE | 335.5 | 167 | 9.6 | 1.4 | 10 |
| 11-HETE | 319.2 | 167.2 | 12.58 | 1.2 | 10 |
| 12(13)EpOME | 295.3 | 195.2 | 13.54 | 1.2 | 7 |
| 12,13-DiHOME | 313.2 | 183.2 | 9.75 | 1.5 | 12 |
| 12-HEPE | 317.2 | 179.2 | 11.54 | 1.23 | 7 |
| 12-HETE | 319.2 | 179.2 | 12.8 | 1.4 | 10 |
| 12-oxo-ETE | 317.2 | 153.1 | 13.05 | 1.2 | 7 |
| 13(14)-EpDPE | 343.2 | 193.2 | 13.71 | 1.2 | 5 |
| 13-HODE | 295.2 | 195.2 | 12.07 | 1.5 | 7 |
| 13-HOTrE | 293.2 | 195.1 | 11.12 | 1.5 | 10 |
| 13-oxo-ODE | 293.2 | 195.1 | 12.48 | 1.24 | 10 |
| 14(15)-EpETE | 317.2 | 207.2 | 12.6 | 1.46 | 5 |
| 14(15)-EpETrE | 319.2 | 219.3 | 13.59 | 1.44 | 5 |
| 14,15-DiHETE | 335.3 | 207.2 | 9.48 | 1.6 | 10 |
| 14,15-DiHETrE | 337.2 | 207.1 | 10.36 | 1.6 | 5 |
| 15(S)-HETrE | 321.2 | 221.2 | 12.98 | 1.47 | 10 |
| 15-deoxy-PGJ2 | 315.2 | 271.2 | 11.36 | 1.1 | 5 |
| 15-HEPE | 317.2 | 219.2 | 11.44 | 1.5 | 5 |
| 15-HETE | 319.2 | 219.2 | 12.21 | 1.51 | 5 |
| 15-oxo-ETE | 317.2 | 113.1 | 12.57 | 1.4 | 7 |
| 16(17)-EpDPE | 343.2 | 233.2 | 13.66 | 1.33 | 5 |
| 16,17 DiHPDA | 361.5 | 233.1 | 10.65 | 1.4 | 8 |
| 17(18)-EpETE | 317.2 | 215.2 | 12.43 | 1.21 | 10 |
| 17,18-DiHETE | 335.3 | 247.2 | 9.14 | 1.77 | 7 |
| 17-HDoHE | 343.2 | 281.2 | 12.29 | 1.46 | 7 |
| 19(20)-EpDPE | 343.2 | 241.2 | 13.39 | 1.1 | 5 |
| 19,20 DiHPDA | 361.5 | 273.1 | 10.35 | 1.4 | 8 |
| 20-COOH-LTB4 | 365.2 | 347.2 | 3.85 | 2.2 | 10 |
| 20-HETE | 319.2 | 275.1 | 11.49 | 1.25 | 7 |
| 20-OH-LTB4 | 351.2 | 195.2 | 4.13 | 1.23 | 12 |
| 5(6)-EpETrE | 319.2 | 191.1 | 14.22 | 1.4 | 5 |
| 5,15-DiHETE | 335.2 | 173.2 | 8.78 | 1.44 | 7 |
| 5,6-DiHETE | 335.2 | 115.2 | 9.03 | 1.4 | 12 |
| 5,6-DiHETrE | 337.2 | 145.1 | 11.45 | 1.2 | 12 |
| 5-HEPE | 317.2 | 115.1 | 11.94 | 1.63 | 5 |
| 5-HETE | 319.2 | 115.1 | 13.13 | 1.2 | 5 |
| 5-oxo-ETE | 317.2 | 273.2 | 13.82 | 1.2 | 5 |
| 6-keto-PGF1a | 369.3 | 163.2 | 3.9 | 2.2 | 7 |
| 6-trans-LTB4 | 335.2 | 195.1 | 8.98 | 1.76 | 7 |
| 7(8)-EpDPE | 343.2 | 113.1 | 13.94 | 1.2 | 5 |
| 8(9)-EpETE | 317.2 | 127.2 | 12.9 | 1.7 | 10 |
| 8(9)-EpETrE | 319.2 | 167.2 | 14.07 | 1.6 | 7 |
| 8,15-DiHETE | 335.2 | 235.2 | 8.73 | 1.4 | 7 |
| 8,9-DiHETE | 335.5 | 126.9 | 9.84 | 1.4 | 12 |
| 8,9-DiHETrE | 337.2 | 127.1 | 11.12 | 1.2 | 12 |
| 8-HEPE | 317.2 | 155.2 | 11.47 | 1.53 | 7 |
| 8-HETE | 319.2 | 155.2 | 12.78 | 1.32 | 5 |
| 9(10)-EpOME | 295.3 | 171.2 | 13.68 | 1.2 | 10 |
| 9,10,13-TriHOME | 329.2 | 171.1 | 5.66 | 1.4 | 8 |
| 9,10-DiHOME | 313.2 | 201.2 | 10.03 | 1.4 | 12 |
| 9,12,13-TriHOME | 329.2 | 211.1 | 5.55 | 1.3 | 8 |
| 9-HETE | 319.2 | 167.2 | 12.96 | 1.26 | 10 |
| 9-HODE | 295.2 | 171.1 | 12.13 | 1.4 | 12 |
| 9-HOTrE | 293.2 | 171.2 | 10.92 | 1.7 | 10 |
| 9-oxo-ODE | 293.2 | 185.1 | 12.74 | 1.24 | 10 |
| d-11-11(12)EpEtrE | 330.2 | 167.2 | 13.74 | 1.4 | 7 |
| d11-14,15-DiHETrE | 348.2 | 207.1 | 10.28 | 1.4 | 12 |
| d4-6-keto-PGF1a | 373.3 | 167.1 | 3.82 | 2.2 | 25 |
| d4-9HODE | 299.2 | 172.3 | 11.94 | 1.4 | 10 |
| d4-LTB4 | 339.2 | 197.2 | 9.26 | 1.2 | 10 |
| d4-PGE2 | 355.2 | 275.3 | 5.91 | 1.6 | 10 |
| d4-TXB2 | 373.3 | 173.2 | 5.6 | 2.8 | 12 |
| d6-20-HETE | 325.2 | 281.2 | 11.44 | 1.2 | 12 |
| d8-5-HETE | 327.2 | 116.1 | 13.05 | 1.1 | 7 |
| LTB3 | 337.2 | 195.2 | 10.5 | 1.32 | 7 |
| LTB4 | 335.2 | 195.1 | 9.1 | 1.23 | 7 |
| LTC4 | 624.3 | 272.1 | 8 | 1.5 | 12 |
| LTD4 | 495.3 | 177.1 | 6.4 | 1.53 | 12 |
| LTE4 | 438.2 | 333.1 | 7.83 | 1.5 | 5 |
| LXA4 | 351.2 | 115.2 | 6.79 | 1.77 | 10 |
| PGB2 | 333.3 | 175.1 | 8.17 | 1.5 | 10 |
| PGD1 | 353.3 | 317.2 | 6.26 | 1.5 | 5 |
| PGD2 | 351.2 | 271.3 | 6.29 | 2 | 10 |
| PGD3 | 349.3 | 269.2 | 5.3 | 1.8 | 5 |
| PGE1 | 353.3 | 317.2 | 6.14 | 1.12 | 5 |
| PGE2 | 351.2 | 271.3 | 5.94 | 2 | 10 |
| PGE3 | 349.3 | 269.2 | 5.1 | 1.8 | 5 |
| PGF2a | 353.2 | 309.2 | 5.69 | 1.8 | 12 |
| PGJ2 | 333.3 | 189.2 | 7.96 | 1.53 | 12 |
| Resolvin D1 | 349.3 | 195.1 | 3.96 | 1.8 | 10 |
| Resolvin E1 | 349.3 | 195 | 3.96 | 1.8 | 10 |
| TXB2 | 369.2 | 169.1 | 5.6 | 2.8 | 5 |
| 10(11)-EpDPE | 343.2 | 153.2 | 13.79 | 1.2 | 5 |

RT: retention time

CE: collision energy to generate product ions

**Supplementary Table 3.** Serum lipid-normalized PBDEs, PCBs and PAHs concentrations (ng/g lipid). Although non-log transformed data are presented, statistical comparisons between postpartum and pregnant women were done on log-transformed data using an unpaired t-test (P-value shown in the last column).

|  | Postpartum (n=120) | Pregnant (n=20) | P-value |  |
| --- | --- | --- | --- | --- |
|  |  |  |  |  |
| PBDE  28 | 45.9 ± 33.4 | 26.3 ± 19.0 | 0.102 |  |
| PBDE 47 | 140 ± 129 | 84.7 ± 62.6 | 0.113 |  |
| PBDE 49 | 36.5 ± 56.4 | 32.3 ± 49.3 | 0.599 |  |
| PBDE 52 | 32.5 ± 46.9 | 16.3 ± 18.6 | 0.285 |  |
| PBDE 66 | 34.2 ± 45.3 | 18.0 ± 25.8 | 0.281 |  |
| PBDE 85 | 98.2 ± 88.3 | 66.6 ± 51.3 | 0.079 |  |
| PBDE 95 | 71.5 ± 68.5 | 45.2 ± 38.7 | 0.469 |  |
| PBDE 99 | 109 ± 94.2 | 68.3 ± 49.3 | 0.233 |  |
| PBDE 100 | 48.3 ± 51.8 | 28.5 ± 20.6 | 0.074 |  |
| PBDE 136 | 2.53 ± 14.9 | N/A | N/A |  |
| PBDE 153 | 41.7 ± 82.4 | 68.9 ± 102 | 0.418 |  |
| PBDE 154 | 15.2 ± 44.0 | N/A | N/A |  |
| PBDE 183 | N/A | N/A | N/A |  |
| PCB 11 | 137 ± 111 | 86.4 ± 81.9 | 0.005 |  |
| PCB 28 | 58.6 ± 39.8 | 30.4 ± 15.6 | <0.001 |  |
| PCB 52 | 106 ± 77.6 | 47.0 ± 27.0 | 0.001 |  |
| PCB 66 | 78.3 ± 62.3 | 37.3 ± 24.1 | 0.003 |  |
| PCB 77 | 12.8 ± 22.9 | 9.47 ± 16.7 | 0.181 |  |
| PCB 84 | 48.4 ± 57.5 | 21.7 ± 13.1 | 0.004 |  |
| PCB 91 | 18.8 ± 38.6 | 6.27 ± 4.30 | 0.001 |  |
| PCB 95 | 90.7 ± 99.7 | 42.1 ± 27.9 | 0.004 |  |
| PCB 101 | 96.9 ± 111 | 43.5 ± 30.4 | 0.006 |  |
| PCB 118 | 67.8 ± 87.2 | 32.8 ± 25.4 | 0.033 |  |
| PCB 126 | 289 ± 382 | 159 ± 167 | 0.119 |  |
| PCB 131 | 14.3 ± 15.8 | 8.35 ± 8.33 | 0.081 |  |
| PCB 132 | 59.4 ± 57.9 | 34.2 ± 21.3 | 0.112 |  |
| PCB 135 | 1.96 ± 13.1 | N/A | N/A |  |
| PCB 136 | 3.01 ± 9.35 | 1.35 ± 3.37 | 0.002 |  |
| PCB 138 | 178 ± 564 | 67.1 ± 48.0 | 0.024 |  |
| PCB 149 | 63.6 ± 70.6 | 31.6 ± 30.1 | 0.008 |  |
| PCB 153 | 19.3 ± 119 | 0.949 ± 3.84 | 0.015 |  |
| PCB 174 | 62.3 ± 73.6 | 48.0 ± 59.3 | 0.362 |  |
| PCB 175 | 8.36 ± 20.7 | 5.13 ± 10.3 | 0.006 |  |
| PCB 176 | 23.9 ± 25.1 | 16.3 ± 20.7 | 0.164 |  |
| PCB 180 | 88.7 ± 87.5 | 56.6 ± 47.2 | 0.167 |  |
| PCB 196 | 116 ± 111 | 62.3 ± 59.8 | 0.161 |  |
| PCB 197 | 26.8 ± 32.6 | 13.6 ± 14.0 | 0.323 |  |
| PCB 202 | 63.0 ± 57.5 | 37.4 ± 36.7 | 0.172 |  |
| Acenaphthylene | 28.7 ± 36.9 | 22.4 ± 28.1 | 0.234 |  |
| Acenaphthene | 120 ± 138 | 73.9 ± 60.7 | 0.532 |  |
| Anthracene | 2,300 ± 1,760 | 1,230 ± 795 | 0.019 |  |
| Benzho-ghi-perylene | 527 ± 1,230 | 219 ± 170 | 0.013 |  |
| Benzo-a-anthracene | 84.8 ± 61.5 | 45.4 ± 27.9 | 0.107 |  |
| Benzo-a-pyrene | 136 ± 137 | 61.6 ± 65.8 | 0.006 |  |
| Benzo-B-flouranthene | 382 ± 316 | 203 ± 204 | 0.039 |  |
| Benzo-ghi-perylene | 881 ± 987 | 384 ± 437 | 0.050 |  |
| Benzo-k-fluoranthene | 178 ± 160 | 93.2 ± 71.8 | 0.342 |  |
| Chrysene | 259 ± 185 | 134 ± 84.2 | 0.052 |  |
| Dibenzo-ah-anthracene | 0.129 ± 1.39 | N/A | N/A |  |
| Fluoranthene | 1,390 ± 1,140 | 647 ± 452 | 0.004 |  |
| Fluorine | 489 ± 448 | 282 ± 185 | 0.095 |  |
| Indo-123-cd-pyrene | 147 ± 214 | 63.9 ± 85.5 | 0.031 |  |
| Mirex | N/A | N/A | N/A |  |
| Phenanthrene | 105 ± 114 | 53.3 ± 41.3 | 0.015 |  |
| Pyrine | 3,250 ± 2,970 | 1,540 ± 1,150 | 0.013 |  |

*****N/A: not available

**Supplementary Table 4.** Serum PBDEs, PCBs and PAHs concentrations (ng/mL of serum). Although non-log transformed data are presented, statistical comparisons between postpartum and pregnant women were done on log-transformed data using an unpaired t-test (P-value shown in the last column).

|  | Postpartum (n=120) | Pregnant (n=20) | P-value |  |
| --- | --- | --- | --- | --- |
|  |  |  |  |  |
| PBDE 28 | 0.128 ± 0.0645 | 0.138 ± 0.0655 | 0.535 |  |
| PBDE 47 | 0.412 ± 0.341 | 0.488 ± 0.379 | 0.212 |  |
| PBDE 49 | 0.112 ± 0.196 | 0.187 ± 0.260 | 0.101 |  |
| PBDE 52 | 0.100 ± 0.173 | 0.0973 ± 0.103 | 0.535 |  |
| PBDE 66 | 0.106 ± 0.152 | 0.107 ± 0.149 | 0.220 |  |
| PBDE 85 | 0.277 ± 0.242 | 0.365 ± 0.245 | 0.229 |  |
| PBDE 95 | 0.206 ± 0.194 | 0.250 ± 0.190 | 0.222 |  |
| PBDE 99 | 0.310 ± 0.270 | 0.389 ± 0.247 | 0.156 |  |
| PBDE 100 | 0.142 ± 0.172 | 0.168 ± 0.124 | 0.246 |  |
| PBDE 136 | 0.00632 ± 0.0347 | N/A | N/A |  |
| PBDE 153 | 0.115 ± 0.189 | 0.353 ± 0.513 | 0.014 |  |
| PBDE 154 | 0.0347 ± 0.0970 | 0.00999 ± 0.0447 | N/A |  |
| PBDE 183 | N/A | N/A | N/A |  |
| PCB 11 | 0.391 ± 0.208 | 0.481 ± 0.457 | 0.539 |  |
| PCB 28 | 0.170 ± 0.0758 | 0.166 ± 0.0578 | 0.916 |  |
| PCB 52 | 0.318 ± 0.230 | 0.254 ± 0.104 | 0.577 |  |
| PCB 66 | 0.233 ± 0.163 | 0.208 ± 0.110 | 0.958 |  |
| PCB 77 | 0.0416 ± 0.0697 | 0.0570 ± 0.0845 | 0.375 |  |
| PCB 84 | 0.151 ± 0.206 | 0.116 ± 0.0583 | 0.895 |  |
| PCB 91 | 0.0608 ± 0.156 | 0.0344 ± 0.0192 | 0.401 |  |
| PCB 95 | 0.280 ± 0.336 | 0.224 ± 0.0967 | 0.965 |  |
| PCB 101 | 0.299 ± 0.376 | 0.242 ± 0.135 | 0.982 |  |
| PCB 118 | 0.204 ± 0.275 | 0.192 ± 0.168 | 0.649 |  |
| PCB 126 | 0.866 ± 1.43 | 0.866 ± 0.811 | 0.576 |  |
| PCB 131 | 0.0428 ± 0.0456 | 0.0460 ± 0.0395 | 0.833 |  |
| PCB 132 | 0.177 ± 0.174 | 0.188 ± 0.106 | 0.299 |  |
| PCB 135 | 0.00550 ± 0.0353 | N/A | N/A |  |
| PCB 136 | 0.0128 ± 0.0427 | 0.00991 ± 0.0252 | 0.270 |  |
| PCB 138 | 0.595 ± 2.32 | 0.380 ± 0.283 | 0.776 |  |
| PCB 149 | 0.202 ± 0.226 | 0.174 ± 0.156 | 0.464 |  |
| PCB 153 | 0.0716 ± 0.484 | 0.00523 ± 0.0203 | 0.075 |  |
| PCB 174 | 0.176 ± 0.179 | 0.255 ± 0.277 | 0.325 |  |
| PCB 175 | 0.0239 ± 0.0552 | 0.0308 ± 0.0618 | 0.859 |  |
| PCB 176 | 0.0686 ± 0.0653 | 0.0848 ± 0.0930 | 0.545 |  |
| PCB 180 | 0.238 ± 0.180 | 0.298 ± 0.168 | 0.078 |  |
| PCB 196 | 0.304 ± 0.212 | 0.327 ± 0.217 | 0.327 |  |
| PCB 197 | 0.0727 ± 0.0740 | 0.0768 ± 0.0658 | 0.457 |  |
| PCB 202 | 0.176 ± 0.139 | 0.194 ± 0.143 | 0.406 |  |
| Acenaphthylene | 0.0770 ± 0.0739 | 0.102 ± 0.0987 | 0.042 |  |
| Acenaphthene | 0.325 ± 0.267 | 0.380 ± 0.191 | 0.101 |  |
| Anthracene | 6.40 ± 3.32 | 6.42 ± 2.10 | 0.571 |  |
| Benzho-ghi-perylene | 1.59 ± 4.96 | 1.20 ± 0.692 | 0.612 |  |
| Benzo-a-anthracene | 0.241 ± 0.117 | 0.253 ± 0.0850 | 0.391 |  |
| Benzo-a-pyrene | 0.357 ± 0.323 | 0.311 ± 0.180 | 0.647 |  |
| Benzo-B-flouranthene | 1.06 ± 0.595 | 1.04 ± 0.538 | 0.688 |  |
| Benzo-ghi-perylene | 2.28 ± 1.91 | 2.03 ± 1.22 | 0.996 |  |
| Benzo-k-fluoranthene | 0.476 ± 0.242 | 0.489 ± 0.231 | 0.356 |  |
| Chrysene | 0.722 ± 0.310 | 0.731 ± 0.229 | 0.478 |  |
| Dibenzo-ah-anthracene | 0.000318 ± 0.00344 | N/A | N/A |  |
| Fluoranthene | 3.87 ± 2.64 | 3.37 ± 0.825 | 0.919 |  |
| Fluorine | 1.35 ± 0.853 | 1.48 ± 0.551 | 0.216 |  |
| Indo-123-cd-pyrene | 0.405 ± 0.693 | 0.375 ± 0.406 | 0.848 |  |
| Mirex | N/A | N/A | N/A |  |
| Phenanthrene | 0.318 ± 0.354 | 0.277 ± 0.132 | 0.760 |  |
| Pyrine | 9.04 ± 7.33 | 8.00 ± 1.94 | 0.879 |  |

*****N/A: not available

**Supplementary Table 5.** Serum free oxylipin concentrations (pmol/mL). Although non-log transformed data are presented, statistical comparisons between postpartum and pregnant women were done on log-transformed data using an unpaired t-test (P-value shown in the last column).

|  | Postpartum (n=120) | Pregnant (n=20) | P-value |  |
| --- | --- | --- | --- | --- |
|  |  |  |  |  |
| 20-COOH-LTB4 | 0.0146 ± 0.0521 | N/A | N/A |  |
| Resolvin E1 | 0.00937 ± 0.0388 | N/A | N/A |  |
| 6-keto-PGF1a | 0.0120 ± 0.0658 | 0.0234 ± 0.0545 | 0.714 |  |
| 20-OH-LTB4 | 0.349 ± 0.391 | 0.231 ± 0.325 | 0.066 |  |
| TXB2 | 76.6 ± 110 | 78.1 ± 78.8 | 0.281 |  |
| PGE3 | 0.0117 ± 0.0683 | 0.0000993 ± 0.000444 | N/A |  |
| PGD3 | 0.107 ± 0.453 | 0.0614 ± 0.139 | 0.868 |  |
| 9,10,13-TriHOME | 9.44 ± 4.12 | 8.86 ± 3.73 | 0.599 |  |
| PGF2a | 0.742 ± 0.807 | 0.574 ± 0.402 | 0.759 |  |
| 9,12,13-TriHOME | 14.0 ± 7.56 | 11.3 ± 5.01 | 0.124 |  |
| PGE2 | 0.708 ± 1.07 | 0.744 ± 0.738 | 0.166 |  |
| PGE1 | 0.0208 ± 0.0502 | 0.00989 ± 0.0239 | 0.861 |  |
| LTD4 | 0.0141 ± 0.0330 | 0.0161 ± 0.0536 | 0.704 |  |
| PGD1 | 0.0171 ± 0.0251 | 0.0183 ± 0.0153 | 0.275 |  |
| PGD2 | 0.103 ± 0.0918 | 0.214 ± 0.278 | 0.018 |  |
| LXA4 | 0.0376 ± 0.0406 | 0.0350 ± 0.0280 | 0.115 |  |
| LTE4 | 0.657 ± 0.897 | 0.450 ± 0.723 | 0.275 |  |
| LTC4 | 0.166 ± 0.564 | N/A | N/A |  |
| PGJ2 | 0.0623 ± 0.0779 | 0.0693 ± 0.0667 | 0.525 |  |
| PGB2 | 0.0840 ± 0.111 | 0.0632 ± 0.0437 | 0.984 |  |
| 8,15-DiHETE | 0.185 ± 0.232 | 0.151 ± 0.282 | 0.599 |  |
| 6-trans-LTB4 | 0.283 ± 0.475 | 0.183 ± 0.199 | 0.760 |  |
| 5,15-DiHETE | 0.0492 ± 0.0565 | 0.0422 ± 0.0301 | 0.568 |  |
| 5,6-DiHETE | N/A | N/A | N/A |  |
| 17,18-DiHETE | 2.64 ± 1.60 | 3.66 ± 3.23 | 0.076 |  |
| LTB4 | 1.43 ± 1.71 | 0.881 ± 1.25 | 0.074 |  |
| 14,15-DiHETE | 0.565 ± 0.433 | 0.680 ± 0.953 | 0.859 |  |
| 11,12-DiHETE | 0.0496 ± 0.0327 | 0.0506 ± 0.0525 | 0.410 |  |
| 12,13-DiHOME | 15.2 ± 15.0 | 11.1 ± 10.9 | 0.054 |  |
| 8,9-DiHETE | 0.0740 ± 0.0832 | 0.0806 ± 0.119 | 0.746 |  |
| 9,10-DiHOME | 15.1 ± 15.2 | 8.28 ± 8.35 | 0.020 |  |
| 19,20-DiHPDA | 1.63 ± 1.00 | 1.97 ± 1.53 | 0.462 |  |
| 14,15-DiHETrE | 0.660 ± 0.229 | 0.559 ± 0.193 | 0.033 |  |
| LTB3 | 0.0453 ± 0.0573 | 0.0335 ± 0.0424 | 0.674 |  |
| 16,17-DiHPDA | 0.201 ± 0.126 | 0.244 ± 0.199 | 0.650 |  |
| 11,12-DiHETrE | 0.594 ± 0.221 | 0.399 ± 0.206 | <0.001 |  |
| 9-HOTrE | 9.37 ± 15.0 | 1.85 ± 1.63 | <0.001 |  |
| 13-HOTrE | 14.4 ± 18.6 | 4.06 ± 3.96 | <0.001 |  |
| 8,9-DiHETrE | 0.286 ± 0.127 | 0.266 ± 0.211 | 0.141 |  |
| 15-deoxy-PGJ2 | 0.443 ± 0.120 | 0.442 ± 0.132 | 0.954 |  |
| 15-HEPE | 0.305 ± 0.394 | 0.180 ± 0.110 | 0.537 |  |
| 20-HETE | 0.961 ± 0.747 | 1.30 ± 0.939 | 0.304 |  |
| 5,6-DiHETrE | 0.367 ± 0.192 | 0.438 ± 0.203 | 0.138 |  |
| 8-HEPE | 0.0739 ± 0.0879 | 0.0622 ± 0.0400 | 0.926 |  |
| 12-HEPE | 14.2 ± 21.8 | 8.14 ± 6.65 | 0.880 |  |
| 5-HEPE | 0.204 ± 0.133 | 0.270 ± 0.201 | 0.083 |  |
| 13-HODE | 131 ± 115 | 79.4 ± 74.3 | 0.013 |  |
| 9-HODE | 75.6 ± 78.0 | 31.0 ± 25.0 | 0.001 |  |
| 15-HETE | 10.3 ± 12.7 | 9.87 ± 7.18 | 0.295 |  |
| 17(18)-EpETE | 0.928 ± 1.31 | 1.15 ± 1.25 | 0.524 |  |
| 13-oxo-ODE | 21.4 ± 13.2 | 9.53 ± 4.10 | <0.001 |  |
| 11-HETE | 5.45 ± 6.98 | 5.79 ± 4.90 | 0.239 |  |
| 15-oxo-ETE | 0.0862 ± 0.0659 | 0.0899 ± 0.0446 | 0.435 |  |
| 9-oxo-ODE | 6.71 ± 5.93 | 2.90 ± 1.53 | <0.001 |  |
| 14(15)-EpETE | 0.0337 ± 0.0391 | 0.0319 ± 0.0409 | 0.532 |  |
| 8-HETE | 2.10 ± 2.01 | 2.10 ± 1.37 | 0.364 |  |
| 12-HETE | 547 ± 584 | 509 ± 389 | 0.280 |  |
| 11(12)-EpETE | 0.0271 ± 0.0294 | 0.0348 ± 0.0322 | 0.335 |  |
| 8(9)-EpETE | 0.227 ± 0.128 | 0.161 ± 0.113 | 0.021 |  |
| 9-HETE | 0.294 ± 0.387 | 0.264 ± 0.150 | 0.572 |  |
| 15(S)-HETrE | 0.830 ± 0.771 | 0.953 ± 0.609 | 0.142 |  |
| 12-oxo-ETE | 0.851 ± 1.44 | 0.888 ± 1.45 | 0.225 |  |
| 5-HETE | 4.48 ± 3.18 | 4.14 ± 3.22 | 0.792 |  |
| 17-HDoHE | 5.67 ± 4.21 | 2.64 ± 1.47 | <0.001 |  |
| 19(20)-EpDPE | 0.404 ± 0.314 | 0.512 ± 0.390 | 0.793 |  |
| 12(13)-EpOME | 27.6 ± 27.7 | 21.5 ± 26.5 | 0.120 |  |
| 14(15)-EpETrE | 0.277 ± 0.164 | 0.207 ± 0.134 | 0.009 |  |
| 9(10)-EpOME | 5.77 ± 4.85 | 5.16 ± 5.09 | 0.248 |  |
| 16(17)-EpDPE | 0.129 ± 0.101 | 0.152 ± 0.146 | 0.883 |  |
| 13(14)-EpDPE | 0.151 ± 0.103 | 0.145 ± 0.130 | 0.108 |  |
| 10(11)-EpDPE | 0.211 ± 0.158 | 0.274 ± 0.246 | 0.486 |  |
| 5-oxo-ETE | 0.231 ± 0.313 | 0.212 ± 0.281 | 0.606 |  |
| 11(12)-EpETrE | 0.404 ± 0.219 | 0.301 ± 0.173 | 0.018 |  |
| 7(8)-EpDPE | 0.390 ± 0.294 | 0.468 ± 0.301 | 0.421 |  |
| 8(9)-EpETrE | 1.03 ± 0.506 | 0.807 ± 0.418 | 0.120 |  |
| 5(6)-EpETrE | 0.975 ± 0.605 | 0.828 ± 0.872 | 0.202 |  |

*****N/A: not available

**Supplementary Table 6.** Serum total oxylipin concentrations (pmol/mL). Although non-log transformed data are presented, statistical comparisons between postpartum and pregnant women were done on log-transformed data using an unpaired t-test (P-value shown in the last column).

|  | Postpartum (n=120) | Pregnant (n=20) | P-value |  |
| --- | --- | --- | --- | --- |
|  |  |  |  |  |
| 20-COOH-LTB4 | 1.26 ± 4.46 | 1.09 ± 0.403 | 0.081 |  |
| Resolvin E1 | 10.7 ± 4.30 | 10.1 ± 3.09 | 0.559 |  |
| 6-keto-PGF1a | 0.0246 ± 0.139 | 0.0835 ± 0.248 | 0.792 |  |
| 20-OH-LTB4 | 0.00842 ± 0.0376 | 0.00740 ± 0.0331 | N/A |  |
| TXB2 | 7.77 ± 11.0 | 7.07 ± 7.75 | 0.531 |  |
| PGE3 | 0.00172 ± 0.00647 | 0.00461 ± 0.0121 | 0.304 |  |
| PGD3 | 0.331 ± 0.851 | 0.237 ± 0.277 | 0.714 |  |
| 9,10,13-TriHOME | 207 ± 680 | 207 ± 445 | 0.608 |  |
| PGF2a | 127 ± 121 | 92.9 ± 92.6 | 0.059 |  |
| 9,12,13-TriHOME | 274 ± 1,030 | 247 ± 532 | 0.683 |  |
| PGE2 | 0.188 ± 0.200 | 0.230 ± 0.207 | 0.420 |  |
| PGE1 | 0.0000695 ± 0.000761 | N/A | N/A |  |
| LTD4 | 0.0200 ± 0.0311 | 0.0187 ± 0.0259 | 0.672 |  |
| PGD1 | 0.0116 ± 0.0399 | 0.0170 ± 0.0363 | 0.710 |  |
| PGD2 | 0.0371 ± 0.102 | 0.0749 ± 0.222 | 0.177 |  |
| LXA4 | 0.497 ± 0.267 | 0.593 ± 0.235 | 0.052 |  |
| LTE4 | 0.0706 ± 0.102 | 0.0487 ± 0.0844 | 0.956 |  |
| PGJ2 | 0.0683 ± 0.0888 | 0.0758 ± 0.0729 | 0.686 |  |
| LTC4 | N/A | N/A | N/A |  |
| PGB2 | 0.835 ± 0.950 | 0.734 ± 0.590 | 0.903 |  |
| 8,15-DiHETE | 0.136 ± 0.294 | 0.382 ± 0.560 | 0.011 |  |
| 6-trans-LTB4 | 0.152 ± 0.108 | 0.151 ± 0.105 | 0.767 |  |
| 5,15-DiHETE | 0.0796 ± 0.0581 | 0.0884 ± 0.0839 | 0.695 |  |
| 17,18-DiHETE | 4.57 ± 1.61 | 3.85 ± 2.00 | 0.005 |  |
| 5,6-DiHETE | N/A | N/A | N/A |  |
| LTB4 | 0.624 ± 0.682 | 0.399 ± 0.434 | 0.206 |  |
| 14,15-DiHETE | 1.30 ± 0.724 | 0.786 ± 0.778 | <0.001 |  |
| 11,12-DiHETE | 0.105 ± 0.0681 | 0.0892 ± 0.0972 | 0.138 |  |
| 12,13-DiHOME | 18.8 ± 17.3 | 16.8 ± 14.7 | 0.675 |  |
| 8,9-DiHETE | 0.155 ± 0.0906 | 0.201 ± 0.133 | 0.050 |  |
| 9,10-DiHOME | 21.4 ± 17.4 | 16.1 ± 11.7 | 0.136 |  |
| 19,20-DiHPDA | 0.959 ± 0.493 | 1.43 ± 0.743 | 0.002 |  |
| 14,15-DiHETrE | 1.04 ± 0.324 | 1.13 ± 0.365 | 0.260 |  |
| LTB3 | 0.152 ± 0.117 | 0.0958 ± 0.0604 | 0.228 |  |
| 16,17-DiHPDA | 0.263 ± 0.114 | 0.506 ± 0.219 | <0.001 |  |
| 11,12-DiHETrE | 1.03 ± 0.270 | 1.54 ± 0.491 | <0.001 |  |
| 9-HOTrE | 5.22 ± 10.3 | 4.41 ± 3.34 | 0.400 |  |
| 8,9-DiHETrE | 3.54 ± 1.06 | 6.46 ± 2.04 | <0.001 |  |
| 13-HOTrE | 5.20 ± 9.43 | 4.78 ± 3.19 | 0.293 |  |
| 15-deoxy-PGJ2 | 4.46 ± 2.20 | 3.05 ± 2.36 | <0.001 |  |
| 15-HEPE | 4.03 ± 1.80 | 3.74 ± 1.48 | 0.371 |  |
| 20-HETE | 27.1 ± 13.6 | 25.3 ± 10.3 | 0.661 |  |
| 5,6-DiHETrE | 18.6 ± 6.04 | 29.1 ± 10.2 | <0.001 |  |
| 8-HEPE | 0.465 ± 0.333 | 0.451 ± 0.597 | 0.087 |  |
| 12-HEPE | 4.61 ± 6.28 | 2.34 ± 1.74 | 0.306 |  |
| 5-HEPE | 6.25 ± 3.63 | 6.46 ± 4.23 | 0.985 |  |
| 13-HODE | 210 ± 193 | 210 ± 158 | 0.690 |  |
| 9-HODE | 97.0 ± 95.2 | 98.1 ± 67.0 | 0.536 |  |
| 15-HETE | 12.7 ± 9.29 | 11.7 ± 6.62 | 0.715 |  |
| 17(18)-EpETE | 496 ± 255 | 304 ± 279 | <0.001 |  |
| 13-oxo-ODE | 47.8 ± 13.9 | 65.3 ± 18.7 | <0.001 |  |
| 11-HETE | 6.25 ± 3.58 | 7.43 ± 4.52 | 0.247 |  |
| 9-oxo-ODE | 16.7 ± 6.89 | 25.1 ± 10.5 | <0.001 |  |
| 14(15)-EpETE | 38.5 ± 27.0 | 23.2 ± 32.1 | <0.001 |  |
| 8-HETE | 3.88 ± 1.68 | 4.19 ± 2.21 | 0.795 |  |
| 12-HETE | 139 ± 190 | 87.2 ± 75.7 | 0.786 |  |
| 15-oxo-ETE | 1.39 ± 0.520 | 1.40 ± 0.646 | 0.645 |  |
| 11(12)-EpETE | 17.0 ± 10.4 | 10.2 ± 10.2 | <0.001 |  |
| 8(9)-EpETE | 31.2 ± 17.9 | 21.8 ± 18.3 | 0.001 |  |
| 9-HETE | 7.08 ± 3.16 | 9.40 ± 6.17 | 0.072 |  |
| 15(S)-HETrE | 3.27 ± 2.02 | 3.92 ± 1.93 | 0.068 |  |
| 12-oxo-ETE | 1.00 ± 0.481 | 1.02 ± 0.476 | 0.900 |  |
| 5-HETE | 9.72 ± 3.94 | 11.4 ± 5.54 | 0.146 |  |
| 17-HDoHE | 19.1 ± 9.69 | 32.9 ± 17.2 | <0.001 |  |
| 19(20)-EpDPE | 65.9 ± 31.6 | 101 ± 48.1 | 0.001 |  |
| 12(13)-EpOME | 631 ± 146 | 717 ± 185 | 0.051 |  |
| 14(15)-EpETrE | 193 ± 61.5 | 181 ± 75.0 | 0.306 |  |
| 9(10)-EpOME | 246 ± 112 | 354 ± 213 | 0.012 |  |
| 16(17)-EpDPE | 25.7 ± 12.7 | 48.9 ± 25.2 | <0.001 |  |
| 13(14)-EpDPE | 33.9 ± 17.4 | 63.9 ± 34.1 | <0.001 |  |
| 10(11)-EpDPE | 56.3 ± 30.6 | 113 ± 68.3 | <0.001 |  |
| 5-oxo-ETE | 0.803 ± 0.401 | 1.21 ± 0.740 | 0.007 |  |
| 11(12)-EpETrE | 162 ± 51.3 | 160 ± 67.2 | 0.612 |  |
| 7(8)-EpDPE | 36.5 ± 17.8 | 63.2 ± 30.6 | <0.001 |  |
| 8(9)-EpETrE | 114 ± 30.7 | 118 ± 41.5 | 0.864 |  |
| 5(6)-EpETrE | 168 ± 66.0 | 182 ± 90.0 | 0.744 |  |

*****N/A: not available

**Supplementary Table 7.** Serum esterified oxylipin concentrations (pmol/mL) determined by subtracting free from total oxylipin values. Although non-log transformed data are presented, statistical comparisons between postpartum and pregnant women were done on log-transformed data using an unpaired t-test (P-value shown in the last column).

|  | Postpartum (n=120) | Pregnant (n=20) | P-value |  |
| --- | --- | --- | --- | --- |
|  |  |  |  |  |
| 20-COOH-LTB4 | 1.24 ± 4.46 | 1.09 ± 0.403 | 0.061 |  |
| Resolvin E1 | 10.7 ± 4.30 | 10.1 ± 3.09 | 0.567 |  |
| 6-keto-PGF1a | N/A | N/A | N/A |  |
| 20-OH-LTB4 | N/A | N/A | N/A |  |
| TXB2 | 1.000 ± 1.09 | N/A | N/A |  |
| PGE3 | 0.00182 ± 0.00669 | 0.00485 ± 0.0124 | 0.315 |  |
| PGD3 | 0.399 ± 0.936 | 0.302 ± 0.280 | 0.780 |  |
| 9,10,13-TriHOME | 197 ± 680 | 198 ± 445 | 0.573 |  |
| PGF2a | 127 ± 121 | 92.3 ± 92.7 | 0.058 |  |
| 9,12,13-TriHOME | 260 ± 1,030 | 236 ± 533 | 0.552 |  |
| PGE2 | 0.230 ± 0.151 | 0.141 ± 0.108 | 0.176 |  |
| PGE1 | N/A | N/A | N/A |  |
| LTD4 | 0.0217 ± 0.0331 | 0.0178 ± 0.0241 | 0.988 |  |
| PGD1 | 0.0291 ± 0.0588 | 0.0385 ± 0.0415 | 0.936 |  |
| PGD2 | 0.238 ± 0.136 | 0.514 ± 0.295 | 0.136 |  |
| LXA4 | 0.460 ± 0.268 | 0.558 ± 0.232 | 0.044 |  |
| LTE4 | 0.109 ± 0.0863 | N/A | N/A |  |
| LTC4 | N/A | N/A | N/A |  |
| PGJ2 | 0.0540 ± 0.104 | 0.0622 ± 0.0588 | 0.388 |  |
| PGB2 | 0.778 ± 0.869 | 0.706 ± 0.590 | 0.946 |  |
| 8,15-DiHETE | 0.199 ± 0.355 | 0.532 ± 0.599 | 0.046 |  |
| 6-trans-LTB4 | 0.0658 ± 0.0493 | 0.0915 ± 0.0761 | 0.376 |  |
| 5,15-DiHETE | 0.0621 ± 0.0426 | 0.104 ± 0.0757 | 0.043 |  |
| 5,6-DiHETE | N/A | N/A | N/A |  |
| 17,18-DiHETE | 2.41 ± 1.54 | 1.50 ± 1.44 | 0.014 |  |
| LTB4 | 0.0694 ± 0.0639 | 0.0811 ± 0.0436 | 0.390 |  |
| 14,15-DiHETE | 0.872 ± 0.632 | 0.559 ± 0.347 | 0.217 |  |
| 11,12-DiHETE | 0.0712 ± 0.0570 | 0.0727 ± 0.0919 | 0.486 |  |
| 12,13-DiHOME | 7.92 ± 15.5 | 7.64 ± 6.62 | 0.123 |  |
| 8,9-DiHETE | 0.121 ± 0.0822 | 0.144 ± 0.0809 | 0.129 |  |
| 9,10-DiHOME | 9.35 ± 12.3 | 8.88 ± 5.43 | 0.404 |  |
| 19,20-DiHDPA | 0.374 ± 0.466 | 0.150 ± 0.181 | 0.609 |  |
| 14,15-DiHETrE | 0.426 ± 0.296 | 0.567 ± 0.413 | 0.511 |  |
| LTB3 | 0.141 ± 0.114 | 0.0938 ± 0.0566 | 0.189 |  |
| 16,17-DiHDPA | 0.101 ± 0.0826 | 0.286 ± 0.184 | <0.001 |  |
| 11,12-DiHETrE | 0.479 ± 0.263 | 1.21 ± 0.484 | <0.001 |  |
| 9-HOTrE | 8.71 ± 21.6 | 2.92 ± 2.61 | 0.820 |  |
| 13-HOTrE | 10.1 ± 24.6 | 2.36 ± 2.17 | 0.888 |  |
| 8,9-DiHETrE | 3.26 ± 1.03 | 6.19 ± 2.04 | <0.001 |  |
| 15-deoxy-PGJ2 | 4.02 ± 2.21 | 2.61 ± 2.38 | <0.001 |  |
| 15-HEPE | 3.73 ± 1.56 | 3.56 ± 1.46 | 0.456 |  |
| 20-HETE | 26.1 ± 13.6 | 24.0 ± 10.3 | 0.569 |  |
| 5,6-DiHETrE | 18.2 ± 6.00 | 28.7 ± 10.1 | <0.001 |  |
| 8-HEPE | 0.395 ± 0.309 | 0.410 ± 0.588 | 0.250 |  |
| 12-HEPE | 2.74 ± 5.71 | N/A | N/A |  |
| 5-HEPE | 6.05 ± 3.57 | 6.19 ± 4.09 | 0.926 |  |
| 13-HODE | 99.3 ± 157 | 130 ± 95.8 | 0.014 |  |
| 9-HODE | 49.7 ± 77.2 | 67.1 ± 48.7 | 0.015 |  |
| 15-HETE | 5.17 ± 3.96 | 4.73 ± 3.14 | 0.921 |  |
| 17(18)-EpETE | 495 ± 255 | 302 ± 279 | <0.001 |  |
| 13-oxo-ODE | 28.2 ± 14.0 | 55.8 ± 18.2 | <0.001 |  |
| 11-HETE | 3.20 ± 2.41 | 3.76 ± 2.89 | 0.950 |  |
| 15-oxo-ETE | 1.31 ± 0.529 | 1.31 ± 0.626 | 0.628 |  |
| 9-oxo-ODE | 10.6 ± 6.11 | 22.2 ± 10.7 | <0.001 |  |
| 14(15)-EpETE | 38.5 ± 27.0 | 23.1 ± 32.1 | <0.001 |  |
| 8-HETE | 2.16 ± 1.51 | 2.35 ± 1.67 | 0.630 |  |
| 12-HETE | N/A | N/A | N/A |  |
| 11(12)-EpETE | 16.9 ± 10.4 | 10.2 ± 10.2 | <0.001 |  |
| 8(9)-EpETE | 31.0 ± 17.9 | 21.6 ± 18.3 | 0.001 |  |
| 9-HETE | 6.78 ± 3.17 | 9.13 ± 6.10 | 0.063 |  |
| 15(S)-HETrE | 2.44 ± 1.57 | 2.97 ± 1.64 | 0.073 |  |
| 12-oxo-ETE | 0.611 ± 0.441 | 0.561 ± 0.398 | 0.901 |  |
| 5-HETE | 5.72 ± 4.08 | 7.27 ± 4.93 | 0.109 |  |
| 17-HDoHE | 13.9 ± 9.24 | 30.2 ± 16.8 | <0.001 |  |
| 19(20)-EpDPE | 65.5 ± 31.6 | 100 ± 47.9 | 0.001 |  |
| 12(13)-EpOME | 604 ± 143 | 696 ± 186 | 0.036 |  |
| 14(15)-EpETrE | 193 ± 61.5 | 181 ± 74.9 | 0.307 |  |
| 9(10)-EpOME | 240 ± 112 | 349 ± 213 | 0.013 |  |
| 16(17)-EpDPE | 25.6 ± 12.7 | 48.8 ± 25.2 | <0.001 |  |
| 13(14)-EpDPE | 33.7 ± 17.4 | 63.7 ± 34.1 | <0.001 |  |
| 10(11)-EpDPE | 56.1 ± 30.6 | 113 ± 68.3 | <0.001 |  |
| 5-oxo-ETE | 0.660 ± 0.400 | 1.06 ± 0.656 | 0.005 |  |
| 11(12)-EpETrE | 161 ± 51.3 | 160 ± 67.2 | 0.615 |  |
| 7(8)-EpDPE | 36.1 ± 17.8 | 62.8 ± 30.6 | <0.001 |  |
| 8(9)-EpETrE | 113 ± 30.7 | 118 ± 41.4 | 0.845 |  |
| 5(6)-EpETrE | 167 ± 65.8 | 181 ± 90.2 | 0.748 |  |

*****N/A: not available

**Supplementary Table 8.** Serum fatty acid and cholesterol concentrations (mg/ml). Although non-log transformed data are presented, statistical comparisons between postpartum and pregnant women were done on log-transformed data using an unpaired t-test (P-value shown in the last column).

|  | Postpartum  (n=120) | Pregnant  (n=20) | P-value |
| --- | --- | --- | --- |
| Tridecylic acid/Tridecanoic acid (C13:0) | 0.162 ± 0.118 | 0.197 ± 0.129 | 0.187 |
| Myristic Acid/Tetradecanoic Acid (C14:0) | 0.0342 ± 0.0390 | 0.0755 ± 0.0524 | <0.001 |
| Palmitic Acid/Hexadecanoic Acid (C16:0) | 0.710 ± 0.540 | 1.55 ± 0.876 | <0.001 |
| Palmitoleic Acid (C16:1) | 0.0482 ± 0.0588 | 0.119 ± 0.0812 | <0.001 |
| Stearic Acid/Octadecanoic Acid (C18:0) | 0.254 ± 0.174 | 0.329 ± 0.177 | 0.031 |
| Oleic Acid/Elaidic Acid (C18:1 n-9 trans/cis) | 0.626 ± 0.505 | 1.43 ± 0.884 | <0.001 |
| Vaccenic acid (C18:1 n-7) | 0.0674 ± 0.242 | 0.0965 ± 0.0603 | <0.001 |
| Linoleic Acid (LA) (C18:2 n-6) | 1.02 ± 0.662 | 1.59 ± 0.903 | 0.001 |
| Linolenic Acid (ALA) (C18:3 n-3) | 0.0241 ± 0.0238 | 0.0504 ± 0.0410 | <0.001 |
| Dihomo-gamma-linolenic acid/8,11,14-eicosatrienoic acid (C20:3 n-6) | 0.0463 ± 0.0330 | 0.0820 ± 0.0464 | <0.001 |
| Arachidonic acid (AA) (C20:4 n-6) | 0.231 ± 0.155 | 0.266 ± 0.127 | 0.125 |
| Eicosapentaenoic acid (EPA) (C20:5 n-3) | 0.0253 ± 0.0217 | 0.0188 ± 0.0264 | 0.029 |
| Docosapentaenoic acid (DPA) (C22:5 n-6) | 0.0249 ± 0.0196 | 0.0405 ± 0.0298 | 0.004 |
| Docosahexaenoic Acid (DHA)/Nervonic acid (C22:6/C24:1) | 0.0439 ± 0.0310 | 0.0900 ± 0.0593 | <0.001 |
| Cholesterol | 0.653 ± 0.548 | 0.908 ± 0.656 | 0.328 |
| Total lipids | 3.97 ± 2.84 | 6.84 ± 3.74 | <0.001 |
| Total fatty acids | 3.32 ± 2.37 | 5.93 ± 3.31 | <0.001 |

**Supplementary Table 9.** Serum fatty acids expressed as percentage of total fatty acids. Data were statistically analyzed by unpaired t-test (P-value shown in the last column).

|  | Postpartum (n=120) | Pregnant (n=20) | P-value |
| --- | --- | --- | --- |
|  |  |  |  |
| Tridecylic acid/Tridecanoic acid (C13:0) | 5.22 ± 2.30 | 3.34 ± 1.18 | 0.001 |
| Myristic Acid/Tetradecanoic Acid (C14:0) | 0.977 ± 0.497 | 1.12 ± 0.738 | 0.034 |
| Palmitic Acid/Hexadecanoic Acid (C16:0) | 21.2 ± 1.81 | 20.4 ± 1.15 | 0.000 |
| Palmitoleic Acid (C16:1) | 1.35 ± 0.547 | 1.15 ± 0.357 | 0.000 |
| Stearic Acid/Octadecanoic Acid (C18:0) | 7.77 ± 0.840 | 8.20 ± 0.713 | 0.000 |
| Oleic Acid/Elaidic Acid (C18:1 n-9 trans/cis) | 18.2 ± 2.70 | 17.6 ± 2.45 | 0.000 |
| Vaccenic acid (C18:1 n-7) | 1.57 ± 1.90 | 1.35 ± 0.202 | 0.984 |
| Linoleic Acid (LA) (C18:2 n-6) | 31.5 ± 3.33 | 32.4 ± 2.80 | 0.000 |
| Linolenic Acid (ALA) (C18:3 n-3) | 0.695 ± 0.294 | 0.682 ± 0.311 | 0.144 |
| Dihomo-gamma-linolenic acid/8,11,14-eicosatrienoic acid (C20:3 n-6) | 1.40 ± 0.336 | 1.35 ± 0.271 | 0.715 |
| Arachidonic acid (AA) (C20:4 n-6) | 7.15 ± 1.28 | 7.28 ± 0.994 | 0.000 |
| Eicosapentaenoic acid (EPA) (C20:5 n-3) | 0.816 ± 0.807 | 0.773 ± 0.229 | 0.006 |
| Docosapentaenoic acid (DPA) (C22:5 n-6) | 0.783 ± 0.306 | 0.809 ± 0.244 | 0.184 |
| Docosahexaenoic Acid (DHA)/Nervonic acid (C22:6/C24:1) | 1.39 ± 0.473 | 1.38 ± 0.384 | 0.099 |

**Supplementary Table 10.** Serum diol/epoxide oxylipins ratios (%). Data were statistically analyzed by unpaired t-test (P-value shown in the last column).

|  | Postpartum (n=120) | Pregnant (n=20) | P-value |
| --- | --- | --- | --- |
|  |  |  |  |
| 17,18,-DiHETE/17,18-EpETE | 1.24 | 2.13 | <0.001 |
| 14,15,-DiHETE/14,15-EpETE | 4.65 | 5.59 | 0.404 |
| 11,12,-DiHETE/11,12-EpETE | 0.692 | 0.914 | 0.021 |
| 12,13,-DiHOME/12,13-EpOME | 3.03 | 1.95 | 0.048 |
| 8,9,-DiHETE/8,9-EpETE | 0.599 | 1.27 | <0.001 |
| 9,10,-DiHOME/9,10-EpOME | 9.29 | 5.79 | 0.076 |
| 19,20,-DiHPDA/19,20-EpDPE | 1.72 | 1.84 | 0.641 |
| 14,15,-DiHETrE/14,15-EpETrE | 0.625 | 0.763 | 0.127 |
| 16,17,-DiHPDA/16,17-EpDPE | 1.27 | 1.33 | 0.754 |
| 11,12,-DiHETrE/11,12-EpETrE | 0.702 | 1.17 | <0.001 |
| 8,9,-DiHETrE/8,9-EpETrE | 3.35 | 5.67 | <0.001 |
| 5,6,-DiHETrE/5,6-EpETrE | 12 | 15.8 | 0.159 |

**Supplementary Figure 1.** Study participants (n=140) were enrolled to the study 7 to 13 months after the Tubbs fire in Northern California in October of 2017. At the time of sampling 120 subjects were postpartum and 20 were pregnant. Of the 120 postpartum subjects, 118 were pregnant when the fire occurred in 2017. Of the 20 pregnant, 8 were pregnant in October of 2017, and 12 conceived shortly thereafter.

**Supplementary Figure 2.** Directed acyclic graph (DAG) generated using DAGitty. Variables with an asterisk were included in the final multilinear regression model.

The DAG illustrates possible links between different confounding variables that could modify the relationship between PM_2.5_ exposure and serum chemical, lipid and oxylipin outcomes. The child’s age indirectly reflects the maternal metabolic state by indicating the duration since childbirth. The direction from wildfire PM2.5 to child’s age indicates that PM2.5 exposure may influence birth outcomes, particularly in preterm infants. Maternal age may modify serum concentrations of PBDEs, PCBs, and PAHs due to alterations in metabolic rate, body fat distribution, and potentially impacting lipid-normalized biomarker levels. This comprehensive framework aims to explore the influence of wildfire PM_2.5_ exposure on maternal and child health, considering relevant confounders based on hypotheses.


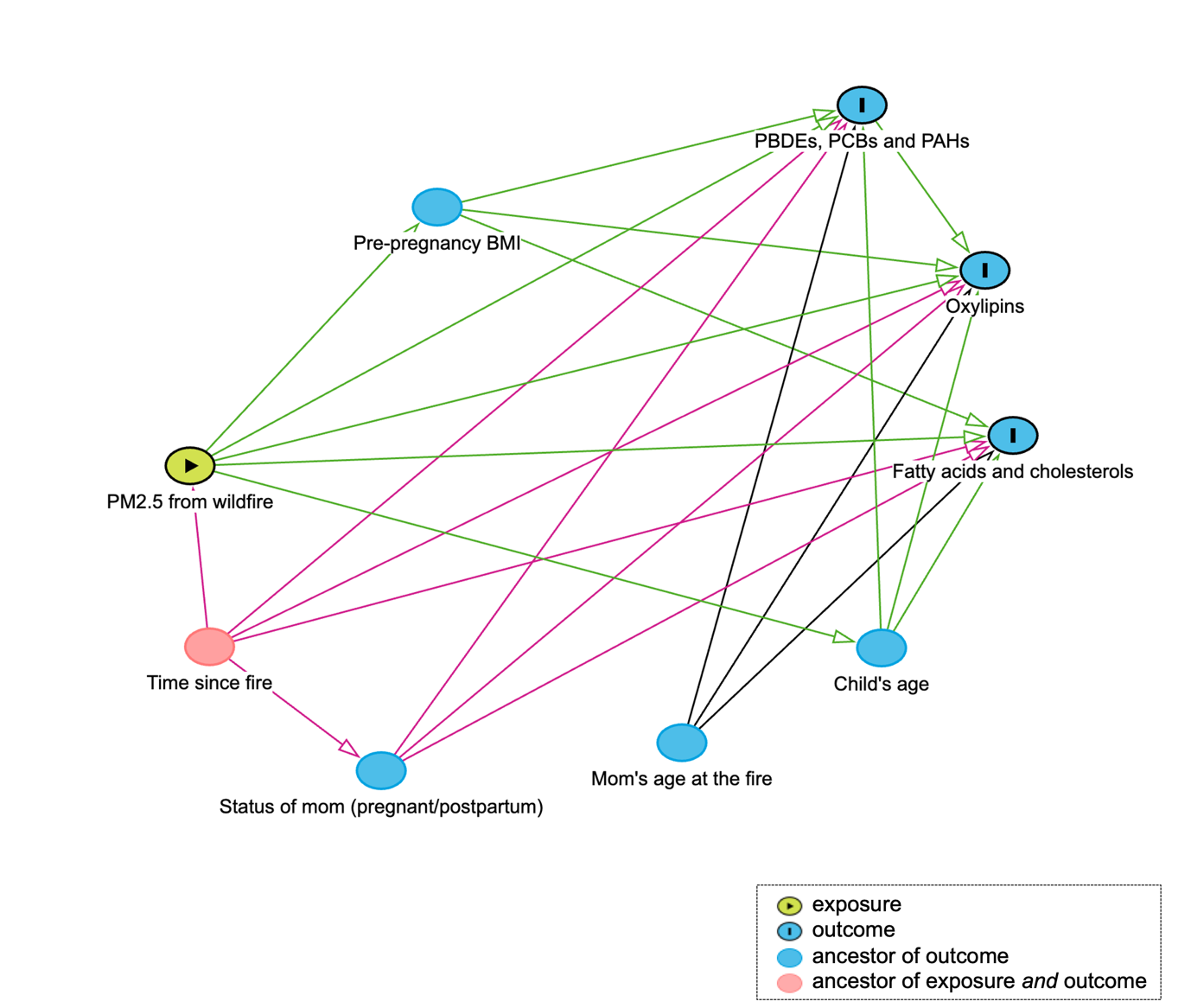


*Abbreviations: PM, particulate matter; BMI, body mass index

**Supplementary Figure 3.** Plot of log-transformed oxylipins and PAH (y-axis) versus PM exposure (x-axis). The graph shows plots for 140 participants.

**Supplementary Figure 4.** Plot of log-transformed oxylipins and PAH (y-axis) versus PM exposure (x-axis). The graph shows plots for the 120 postpartum participants.
