# Supplementary material for: Gestational exposure to particulate matter from urban wildfires is associated with changes in circulating oxylipins but not flame retardants 7 to 13 months post-exposure
Source: Environ Int. Author manuscript; Available in PMC 2026 May 26. (PMC13201207; doi:10.1016/j.envint.2025.109468)
Supplement: 2 [file NIHMS2174401-supplement-2.docx]

**Supplementary Methods**

## Extraction of PBDEs, PCBs and PAHs from Serum

Serum samples stored at -80°C were thawed on ice for 2 h before extraction. An aliquot of 0.5 mL serum was added into a disposable glass tube (Cat # 14-961-25) on ice, spiked with 10 µL of surrogate standard mix solution containing 500 ng/mL ^13^C_12_-PCB-11, ^13^C_12_-PCB-97, ^13^C_12_-BDE-28, ^13^C_12_-BDE-118, and D_12_-Chrysene in isooctane (AccuStandard, Inc., New Haven, CT, USA), and mixed with 0.5 mL of formic acid. The mixture was ultrasonicated for 10 min at room temperature, and then applied to Oasis HLB SPE column which was preconditioned with two aliquots of 3 mL methanol and two aliquots of 3 mL Millipore water /formic acid/methanol mixture (v/v/v, 94.5/0.5/5). The columns were washed with two aliquots of 3 mL Millipore water /formic acid/methanol mixture (v/v/v, 94.5/0.5/5), and dried under vacuum (−5 mmHg) for 5 min. Sep-Pak SPE columns were then placed underneath the Oasis HLB SPE columns, and the analytes were eluted with three aliquots of 3 mL dichloromethane under vacuum (−10 mmHg) into disposable glass tubes (Cat # 14-961-27). The extracts were dried under nitrogen, reconstituted in 100 µL of isooctane fortified with 25 ng/mL mirex, sonicated for 2 min, and transferred into GC/MS vials with inserts for gas chromatography-electron ionization- tandem mass spectrometry (GC-EI-MS/MS) analysis.

## Total lipids (Fatty Acids and Cholesterol) and total oxylipin analysis

Total lipids were extracted from human serum samples using the Folch method with a minor modification (Folch, Lees, & Stanley, 1957). Briefly, serum samples were stored at -80°C and were thawed on ice for 1.5-2 h before extraction. An aliquot of 250 µL serum was added into an 8 mL glass tube containing 350 µL of chilled 1 mM 2Na-EDTA/0.9% KCl and 2.4 mL of chloroform/methanol (2:1 v/v) with 0.002 % BHT. The sample was vortexed for 20 s, and centrifuged at 2,000 rpm at 0°C for 10 min on a Beckman Coulter centrifuge (rotor-GH3.8A). The bottom layer was transferred to a new 8 mL glass tube. To the remaining upper layer, 1.6 mL of cold chloroform was added. The mixture was vortexed for 10 s, centrifuged at 2,000 rpm at 0°C for 10 min, and the bottom layer was transferred to the 8 mL glass tube containing the first chloroform extract. The combined bottom chloroform layers were dried under nitrogen, reconstituted in 2 mL of chloroform/methanol (2:1 v/v), vortexed, transferred to 2 mL GC amber vials, and stored at -80°C until further use.

A portion (1 mL) of the 2 mL Folch extracts was used for total fatty acid and cholesterol determination. Samples were transferred to dried kimble test-tubes pre-washed with chloroform/methanol (2:1 v/v). Internal standards consisting of 50 µL of 5-α Cholestane (0.125 mg/mL in 2:1 chloroform/methanol) and 25 µL of PC-17 (3 mg/mL in 2:1 chloroform/methanol) were added to the kimble test-tubes. Then, 0.4 mL of toluene, 3 mL of methanol, and 600 µL of 37% HCl in methanol was added to each sample. Samples were vortexed and heated on a heating block at 90°C for 60 min. After cooling the samples at room temperature for 4-5 min, 1 mL of hexane and 1mL of distilled water were added. The samples were vortexed and allowed to sit for ~15 minutes at room temperature for the hexane layer containing the transesterified fatty acids to separate from the rest of the aqueous phase. The upper hexane layer (around 900 µL) was transferred to a 1.5 mL centrifuge tube containing 450 µL of distilled water. The mixture was vortexed, and centrifuged at 5,000 rcf for 2 min at room temperature. The upper hexane layer was transferred to a new 1.5 mL centrifuge tube, evaporated under nitrogen, and reconstituted in 100 µL of hexane. Samples were vortexed and transferred to GC vials with inserts, and stored in a -80°C freezer until analysis.

Total oxylipin analysis was performed on the remaining 1 mL portion of the 2 mL Folch extract. The sample was transferred to 2 mL polypropylene centrifuge tubes and dried under nitrogen. The following mixtures were then added to each sample: 1) 10 μL of antioxidant solution containing 0.2 mg/mL BHT, EDTA, and triphenylphosphine (TPP) in water/methanol (1:1 v/v); 2) 10 µL of surrogate standard solution containing 2 μM of d11-11(12)-EpETrE, d11-14,15-DiHETrE, d4-6-keto-PGF1α, d4-9-HODE, d4-LTB4, d4-PGE2, d4-TXB2, d6-20-HETE and d8-5-HETE in methanol; 3) 200 μL of methanol containing 0.1% BHT and 0.1% acetic acid; and 4) 200 μL of 0.4 M sodium hydroxide in methanol/water (1:1 v/v). The mixture was vortexed, heated on a heating block at 60°C for 30 min, and cooled down at room temperature for approximately 5 min. Then, 25 μL of acetic acid and 1575 μL of water were added to each sample. This mixture was vortexed and loaded onto 60 mg Waters Oasis HLB 3cc SPE columns (Waters, Milford, MA, USA) prewashed with one column volume of ethyl acetate and two columns volume of methanol, and pre-conditioned with two column volumes of SPE buffer containing 5% methanol and 0.1% acetic acid in ultrapure Millipore water. The columns were rinsed with two column volumes of SPE buffer and dried under vacuum (≈20 psi) for 20 min. Total oxylipins were eluted with 0.5 mL of methanol and 1.5 mL of ethyl acetate into 2 mL centrifuge vials. The samples were dried under nitrogen, reconstituted in 100 µL of LCMS methanol, and stored at -80° until UPLC-MS/MS analysis.

*Extraction of Free Oxylipins from Serum*

Serum was vortexed and a 200 µL aliquot was transferred to ice-cooled 2 mL polypropylene tubes containing 10 µL of 2µM surrogate spike solution containing d11–11(12)-epoxy-eicosatrienoic acid (d11-11(12)-EpETrE), d11–14,15-dihydroxyeicosatrienoic acid (d11–14,15-DiHETrE), d4-6-keto Prostaglandin F1α (d4–6-keto-PGF1α), d4- 9-hydroxyoctadecadienoic acid (d4–9-HODE), d4-Leukotriene B4 (d4-LTB4), d4-prostaglandin E2 (d4-PGE2), d4-thromboxane 2 (d4-TXB2), d6-20-hydroxyeicosatetraeonic acid (d6–20-HETE) and d8-5-hydroxyeicosatetraeonic acid (d8–5-HETE) in methanol. Each sample received 600 µL of pre-mix solution containing 0.002% BHT, 250 µM EDTA, and 0.01% acetic acid in methanol/MiliQ water mixture (1:4 v/v). Samples were vortexed for approximately 5 s and centrifuged at 0°C for 10 min at 5,000 rcf (13,000 rpm on a microcentrifuge). Free oxylipins were purified from the supernatant using 100 mg tC18 Sep-Pak solid phase extraction (SPE) columns (Waters Corp) prewashed with 1.5 mL of methanol and equilibrated with 1.5 mL of 20% methanol in water. The entire supernatant was loaded onto the column (one sample per column), which was washed with 1.5 mL of 20% methanol and 1.5 mL of hexane. Oxylipins were eluted into 2 mL polypropylene tubes (pre-rinsed with methanol) with 2 mL of methanol. The extract was dried under nitrogen, reconstituted with 100 µL of LC-MSMS grade methanol, vortexed for 10 s, and transferred into 2 mL tubes with 0.1 µm centrifugal Duropore filter (Millipore). Sample was centrifuged at 0°C for 2min with 5,000 rcf. The filtered samples were transferred into 2 mL autosampler vials with 150 µL inserts and caps and stored at -80°C freezer until UPLC-MS/MS analysis.
